# Mitigating greenhouse gas emissions and enhancing composting efficiency using biochar, used oil, and compost inoculum amendments

**DOI:** 10.1038/s41598-025-21144-w

**Published:** 2025-10-07

**Authors:** Azza A. Mohammed, Samy M. Younis, A. E. Ghaly, Mohamed M. Ibrahim

**Affiliations:** Department of Agricultural Engineering, Faculty of Agriculture, Cairo University, Giza, 12613 Egypt

**Keywords:** Used cooking oil, Biochar, GHGs emission, Compost maturity, Microbial pathogens, Biotechnology, Environmental sciences, Microbiology

## Abstract

This study aimed to evaluate the effectiveness of combining biochar, used cooking oil, and compost inoculum as amendments to improve the composting process of agricultural residues (broccoli and pepper waste) and manure. The objective was to enhance compost maturation, microbial safety, and environmental performance, particularly in terms of greenhouse gas emissions. Six treatments were tested: 50% manure + 50% agricultural residues (T_1–1_, control), 60% manure + 40% agricultural residues (T_1– 2_, control), 50% manure + 50% agricultural residues + 20 ml used cooking oil (T_2–1_), 60% manure + 40% agricultural residues + 20 ml used cooking oil (T_2–2_), 50% manure + 50% agricultural residues + 20% inoculum compost (T_3–1_), and 60% manure + 40% agricultural residues + 20% inoculum compost (T_3–2_), Then the first experiment was conducted to produce compost without adding biochar. After the first experiment was completed, a second experiment was conducted with the addition of 10% biochar at the same proportions as in the first experiment. The resulting mixture was composted in a controlled reactor with aeration and mixing. Results showed that the combination of used cooking oil and biochar significantly increased temperature, prolonged the thermophilic phase, and reduced the composting period to 10 days. This combination also decreased GHG emissions by 27–33% and eliminated methane emissions. Pathogenic bacteria (*E. coli* and Salmonella) were fully removed within one week. The findings suggest that integrating these amendments can accelerate composting, improve hygienic quality, and reduce environmental impacts.

## Introduction

 Due to the significant population growth and industrial and agricultural development, agricultural waste has increased, including residues from greenhouses, gardens, and farms. Recycling is an important strategy for obtaining energy and organic products from underutilized resources^1^. The increased demand for food protein has led to increased livestock production and consequently increased livestock manure production. Improper disposal of manure can result in indirect agricultural pollution through gaseous emissions^2^. Researchers indicate that methane and nitrous oxide emissions from animal husbandry contribute to 80% of greenhouse gas emissions with carbon dioxide accounting for about 12%. Therefore, environmentally friendly recycling methods are needed to transform manure into stable finished products through composting, as manure is a valuable fertilizer resource rich in organic substances and nutrients like N, P, K^2,3^. Although composting is a natural biochemical process, it can have negative effects such as the emission of hazardous gases like carbon dioxide, methane, nitrous oxide, and ammonia due to chemical reactions within the organic waste itself during mineralization and decomposition^4,5^. Various volatile organic compounds are formed during the composting process, and the rates and forms of gas emissions depend on the feedstock and aeration materials^6^. Compost, as an organic fertilizer, should provide sufficient nutrients to crops. However, carbon and nitrogen nutrients are lost through gas emissions during production^7^. Ammonia (NH_3_) and nitrous oxide (N_2_O) emissions contribute to nitrogen loss by 79–94% and 9.2–9.7% respectively, while methane emissions result in 1.85% carbon loss^8^. Organic carbon breaks down to provide energy for microbial activity and is mainly released as carbon dioxide (CO_2_), while organic nitrogen is converted by microorganisms into ammonium nitrogen, which is released as ammonia (NH_3_) during composting^9,10^. The accumulation of greenhouse gases (CO_2_, CH_4_, N_2_O) in the atmosphere is the primary driver of global warming. This phenomenon poses a significant threat to global ecosystems by causing climate change, which is characterized by rising global temperatures, sea-level rise, and an increased frequency of extreme weather events, all of which profoundly impact biological activity and food security^3^. Global warming poses a significant threat to all organisms and has become a major global concern due to rapid economic development. Low nitrogen content in organic waste can prolong maturation time and reduce compost quality. To address this issue, adding substrates such as biochar with a high nitrogen ratio can help improve compost quality^11^. Biochar is an insoluble solid material produced through the pyrolysis of organic raw materials at high temperatures and limited oxygen supply^12^. Biochar has stable properties and can serve as a filler during compost production, providing an ideal environment for microorganisms due to its high porosity and exchange cations capacity. It can help reduce compost maturation time, improve quality, and lower greenhouse gas emissions^13,14^. The efficacy of biochar in improving the composting process stems from its unique physicochemical properties. Its highly porous structure and large specific surface area provide a physical refuge for beneficial microorganisms, protecting them from environmental stresses and promoting colonization^15^. Furthermore, biochar can directly mitigate gaseous losses through the adsorption of volatile compounds like ammonia (NH_3_) onto its surface. Its high cation exchange capacity (CEC) allows it to retain essential nutrient cations, particularly ammonium (NH_4_^+^), thereby reducing nitrogen loss via volatilization and enhancing the nutrient content of the final compost^16^. This multi-faceted mechanism not only curtails greenhouse gas emissions but also contributes to a more stable and nutrient-rich end product.

In addition to biochar, other carbon-rich amendments can be utilized to optimize the composting C/N ratio. Used cooking oil (UCO), a common organic waste, can serve as a potent, energy-dense carbon source. Its high lipid content provides substantial energy for microbial metabolic activities, which can help accelerate the decomposition of organic matter and potentially enhance the thermophilic phase of composting, a critical stage for pathogen inactivation^17^. However, the application rate of UCO must be carefully calibrated, as excessive amounts may create anaerobic pockets, leading to the production of phytotoxic compounds and unpleasant odors. Therefore, its co-application with a porous material like biochar could potentially mitigate these risks while harnessing its energetic benefits.

To further accelerate and guide the decomposition process, the use of a compost inoculum is a promising strategy. Compost inoculums are microbial consortia containing pre-selected, highly efficient microorganisms, such as cellulolytic fungi (e.g., Trichoderma sp.) and thermophilic bacteria (e.g., Bacillus sp.). These inoculants can significantly shorten the composting period by rapidly breaking down complex organic polymers like cellulose and lignin^18^. By introducing a robust and targeted microbial community from the outset, it is possible to steer the composting process towards more efficient mineralization pathways, potentially outcompeting native microbial populations that may be responsible for higher emissions of CH_4_ and N_2_O.

The addition of used oil to biochar systems can delay the onset of anaerobic conditions through a lipid-mediated modification of biochar’s porous architecture. Lipids in used oil primarily triglycerides, free fatty acids, and other hydrophobic hydrocarbons adsorb to biochar pore surfaces, reducing wettability and limiting water saturation in micro and mesopores^19^. This preserves air-filled channels for oxygen diffusion, a key factor in preventing anaerobic microsite formation. Simultaneously, these lipids provide a high-energy carbon source for aerobic microbes, stimulating growth and respiration that help maintain oxidizing conditions^20^.

While the individual effects of biochar or microbial inoculants on composting are well documented, there is a significant research gap regarding the synergistic effects of combining biochar with used oil and a compost inoculum. The interactions between a porous sorbent (biochar), an energy-rich substrate (used oil), and a specialized microbial community (inoculum) have not been comprehensively studied in compost production. This process embodies a three-pronged synergy: oil lipids create the pore environment, biochar provides an aerated environment, and aerobic microbes utilize the lipid carbon to outcompete anaerobes. Documented benefits include improved composting performance, reduced methane emissions, and stabilized aerobic biodegradation. Understanding how these modifiers interact is critical to developing an optimal, multifaceted strategy to simultaneously reduce greenhouse gas emissions and enhance the agronomic value of the final compost. Therefore, this study aims to evaluate the combined effect of these three modifiers on composting efficiency and gas emissions.

## Materials and methods

### Collection and preparation of raw materials

**Fig. 1:**
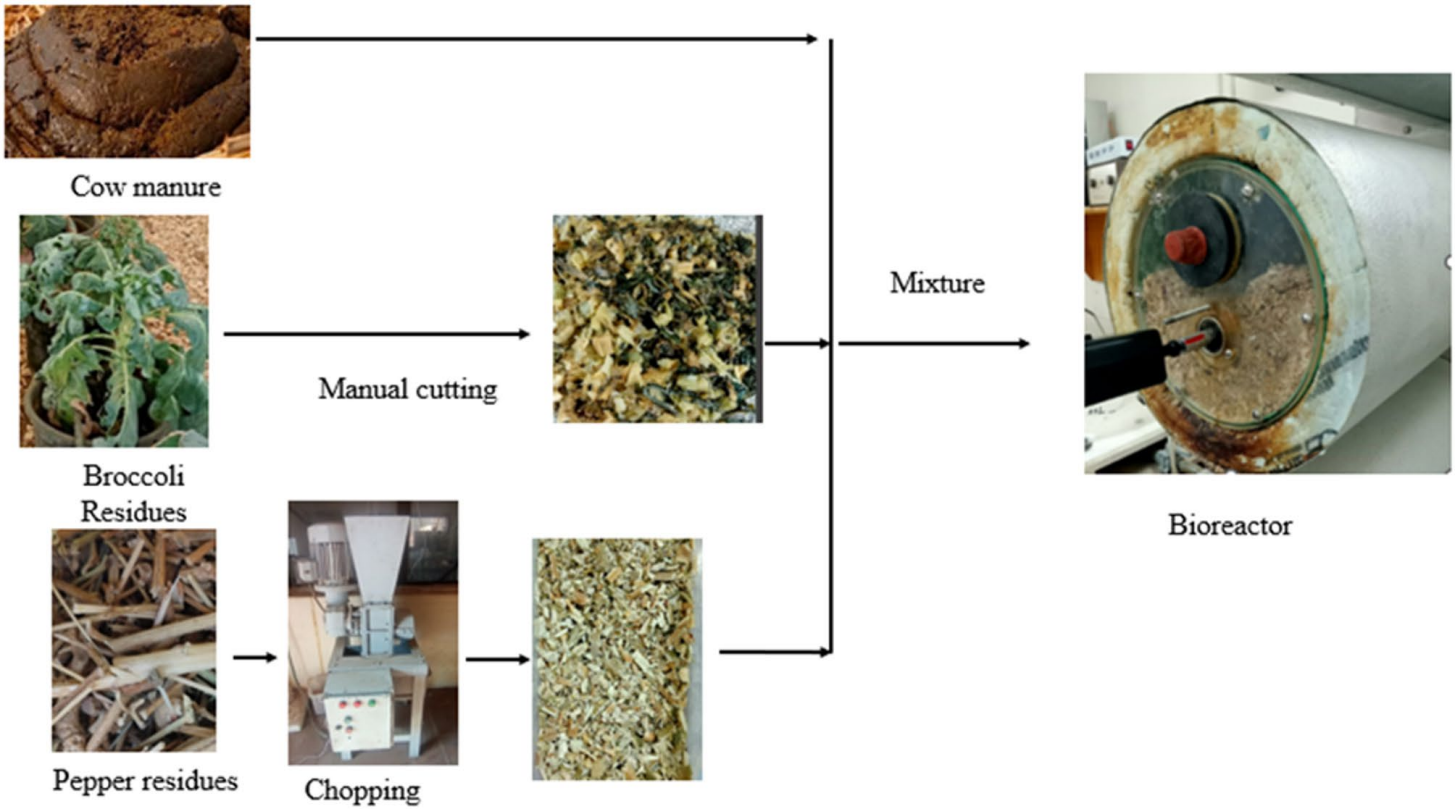
Raw materials used for compost production.

Pepper and broccoli residues (stem and leaves) were collected from greenhouses and used as agricultural plant waste. They were cut and ground using a grinding machine to a size of 5–6 mm to reduce the particle size in order to enhance the microbial decomposition process (Fig. 1). Sawdust and cow manure were obtained from Cairo University’s Faculty of Agriculture research farm.

#### Biochar preparation

The type of raw materials used, pyrolysis temperature and process time are the main factors that control the physical and chemical properties of the biochar produced. Biochar was prepared from the same residues used to produce compost with the same mixing ratios (pepper, sawdust and manure). It was sufficient to use one plant residue as a biosolid material which can stimulate the growth of nitrogen removers and reduce NH_3_ emissions, and manure as an animal waste characterized by a high nitrogen content. The second plant waste ratio was replaced by sawdust as a woody biomass characterized by a high surface area due to its high lignin content, and also forms more organic layers to provide a food shelter for microbes and improve microbial activities.

**Fig. 2:**
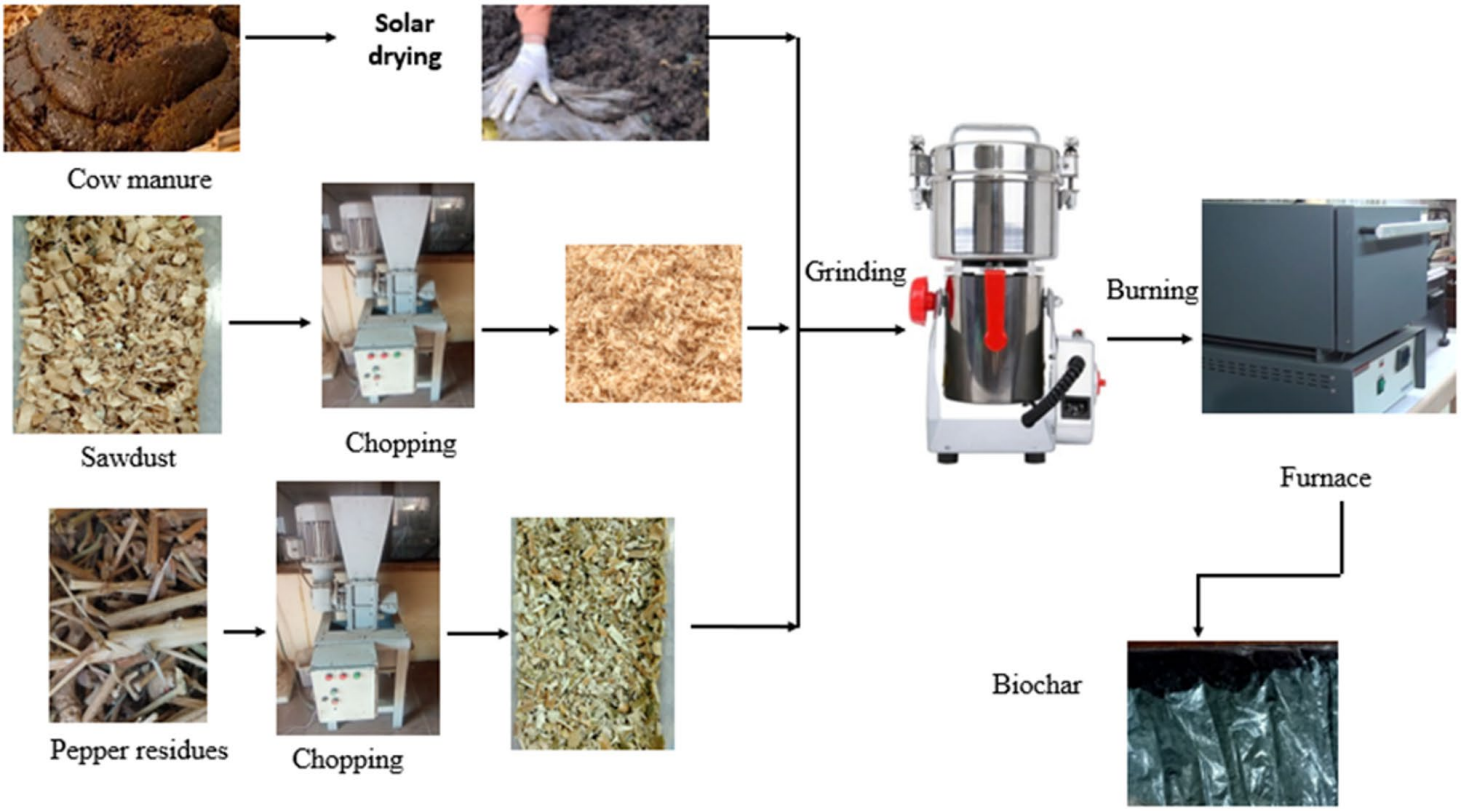
Raw materials used for biochar production.

As shown in Fig. 2, the manure was spread out on the ground for two weeks, it was constantly agitated until it dried and then ground to a small size. Pepper and sawdust were chopped and ground, then biochar was produced separately from each waste (pepper residues, sawdust, and manure) individually by subjecting them to pyrolysis at a controlled temperature 550 °C for 3 h under limited oxygen conditions using a sealed muffle furnace with a vented exhaust to allow gas escape without air intrusion. Each type of feedstock was pyrolyzed separately to maintain consistency in derived biochar properties, then the resulting biochar’s were then mixed according to the original green waste mixing ratios used in each treatment.

and then used as a stable carbon material^21^.

Chemical and physical properties of the waste and biochar were measured in the Biological and Environmental Engineering System Laboratory, Department of Agricultural Engineering and the Laboratory Complex, Faculty of Agriculture, Cairo University, Egypt, such as pH, electrical conductivity, moisture content, volatile solid, ash, total nitrogen, total carbon, calcium, potassium, sodium, phosphorus, iron, manganese, and zinc as demonstrated in Tables 1 and 2. All raw material measurements were performed in triplicate (*n* = 3).

**Table 1:** Chemical properties of different agricultural and animal residues.

Parameters	Pepper	Broccoli	Sawdust	Manure	Inoculum compost	Used oil
pH	5.84	5.18	8.2	7.44	7.1	5.9
Electrical Conductivity (ds m^−1^)	0.64	0.61	0.68	1.15	2.9	–
Moisture content (%)	9	10.75	9.04	66.7	39	–
Volatile solid (%)	85.10	87.20	98.91	77.9	86.4	99.92
Ash (%)	14.90	12.80	1.09	22.10	13.6	0.08
Total Nitrogen (%)	1.82	0.27	0.31	2.03	1.4	0.035
Total Carbon (%)	51.77	47.44	57.54	43.64	30	70.76
Ca (%)	0.32	0.56	0.04	0.56	1.2	0.006
K (%)	2.18	4.20	0.03	1.80	0.8	0.002
Na (%)	0.15	0.18	0.11	0.28	0.57	0.03
P (%)	0.22	0.29	0.28	2.32	0.26	0.001
Fe (mg kg^−1^)	24.15	35.84	20.91	187.95	–	–
Mn (mg kg^−1^)	32.33	33.60	32.40	111.95	–	–
Zn (mg kg^−1^)	25.47	18.16	2.40	212.14	–	–
Total oil and grease (%)	–	–	–	–	–	90.56
Carbohydrates (%)	–	–	–	–	–	7.8

“–” means “undetected”.

**Table 2:** Chemical properties of the biochar.

Parameters	Pepper	Sawdust	Manure
pH	9.8	8.1	9.2
Electrical Conductivity (ds m^−1^)	6.5	0.9	6.29
Volatile solid (%)	64	81.8	64.8
Ash (%)	36	18.2	35.2
Total Nitrogen (%)	0.45	0.21	0.54
Total Carbon (%)	62.77	45.6	58.82
K (%)	1.08	0.23	0.89

### Experimental system

**Fig. 3:**
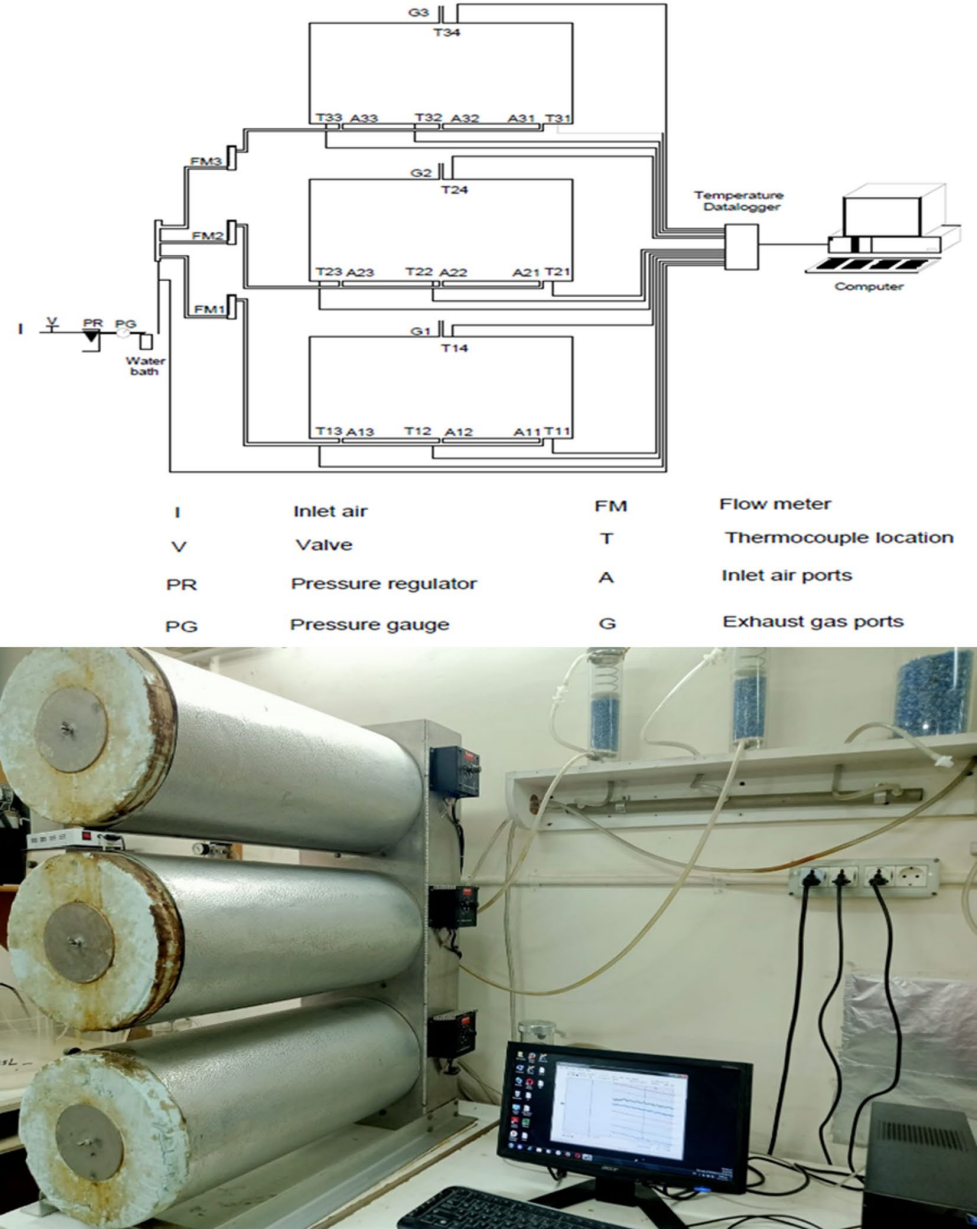
Experimental system.

Practical experiments were conducted in the Biological and Environmental Engineering System Laboratory at the Faculty of Agriculture - Cairo University, Egypt. The experiment was carried out inside a fully equipped reactor with a controlled agitation unit and a forced ventilation source as shown in Fig. 3. The device comprised three bioreactors and a data collection unit. Each bioreactor provided a size ranging from 3 to 3.5 kg of compost mixture, plus 25% size as a headspace. Each bioreactor was designed with an inner diameter of 203 mm, a length of 520 mm, and a thickness of 5 mm. The bioreactors were covered with a removable circular glass sheet with a diameter of 203 mm and a thickness of 6 mm. Inside each bioreactor, a stainless-steel column was used for agitation, rotating at a rate of 5 rpm. Air was continuously supplied to the reactor from an air source passing through a pressure regulator and a pressure gauge to maintain a pressure of about 5 kPa. The air then passed through a water bath to humidify it before entering the reactor to adjust the humidity level, with an air flow rate set at approximately 0.15 m^3^ h^−1^.

Each bioreactor was equipped with four thermocouples for temperature measurements, three located in the lower part to measure the compost mass temperature, and one placed in upper part near the outlet air hole to measure the exhaust gas temperature. The data acquisition unit included a main unit, a scanning card, software, temperature sensors, and a computer. The main unit (Multiscan 1200, Omega, Stamford, CT) was connected to the computer via the RS 232 interface. The thermal scanning card had 24 differential input channels, with each channel programmable to receive data from the thermocouples.

### Experimental setup

Plant residues of 3.5 kg were mixed with animal waste (manure) in two different ratios 50%:50% and 60%:40%, and the mixing ratio of pepper to broccoli was 1:1.5 based on dry weight as shown in Table 3. Each treatment was conducted in triplicate (*n* = 3).

Used cooking oil (20 ml) was added to each mixture continuously every 24 h as a bioavailable carbon source, and 20% of animal wastes were added to each mixture as inoculum after studying some of their physical and chemical properties as shown in Table 1 to study its effect on temperature and compost maturation period.

After completing the first composting experiment using six different treatments, a second experiment was conducted in which 10% biochar was added to the same treatments. These modified treatments were then composted to assess the effect of biochar addition on temperature and gas emissions, by comparing the results before and after its incorporation.

The moisture content of these mixtures was determined and calculated using Eq. (1) and adjusted to 50–60%, and the C:N ratio for each mixture was determined using Eq. (2) and adjusted to about 30:1 by adding urea (46% nitrogen) as a nitrogen supplement.

The final mixture used in each experiment was mixed well, and then placed in the bioreactor occupying 75% of its total volume. The mixing unit was then operated at 5 rpm, the system operated at a constant aeration rate of 0.15 m^3^ h^−1^ during all experiments, and temperatures of bioreactors were monitored continuously.

1 $${\text{M}} = \frac{{{\text{Wet~Weight}} - {\text{Dry~weight}}}}{{{\text{Wet~Weight}}}}~ \times 100$$

where M is moisture content, wet basis, %.

2 $${\text{C}}:{\text{N}}\;{\text{ratio}} = \frac{{{\text{Q}}1({\text{C}}1\left( {100 - {\text{M}}1} \right) + {\text{Q}}2({\text{C}}2\left( {100 - {\text{M}}2} \right) + \ldots }}{{{\text{Q}}1({\text{N}}1\left( {100 - {\text{M}}1} \right) + {\text{Q}}2({\text{N}}2\left( {100 - {\text{M}}2} \right) + \ldots }}$$

where Q_1_ is quantity of material (1); C_1_ is percentage of carbon in material (1); M_1_ is moisture content of material (1); N_1_ is percentage of nitrogen in material (1); Q_2_ is quantity of material (2); C_2_ is percentage of carbon in material (2); M_2_ is moisture content of material (2); N_2_ is percentage of nitrogen in material (2).

**Table 3:** The experimental treatments without and with adding biochar.

Treatments	Mixing ratios (%)		Symbol
	Manure: Agricultural residues	Manure: Broccoli: Pepper	
Before adding biochar			
Control	50:50	50:20:30	T_1–1_
	60:40	60:16:24	T_1–2_
Adding 20 ml of used cooking oil	50:50	50:20:30	T_2–1_
	60:40	60:16:24	T_2–2_
Adding 20% of compost	50:50	50:20:30	T_3–1_
	60:40	60:16:24	T_3–2_
After adding biochar			
Control + 10% biochar	50:50	50:20:30	T_1–1_
	60:40	60:16:24	T_1–2_
Adding 20 ml of used cooking oil + 10% biochar	50:50	50:20:30	T_2–1_
	60:40	60:16:24	T_2–2_
Adding 20% of compost + 10% biochar	50:50	50:20:30	T_3–1_
	60:40	60:16:24	T_3–2_

### Physicochemical characterization and microbiological

In distilled water (extract), pH and electrical conductivity (EC) were measured by 1:5 ratio, by taking 10 g of residue and adding it to 50 ml of deionized water. The measurements were taken in the extract after filtration using pH and EC electrodes^22,23^. Moisture content of all residues was calculated on a wet basis from a 250 g sample dried at 105 ± 5 °C for 24 h until a constant weight was obtained. Moisture content (%) was calculated as the mass loss due to water removal (g g^−1^) divided by the moisture.

Volatile solids are the organic matter (largely carbon, oxygen and nitrogen) that are lost at 550 °C, leaving only the ash. Approximately 50 g of representative composite samples were taken on a wet basis for each treatment, dried, and ground for total nitrogen (TN) and total carbon (TC) analysis. The analysis was done using kjeldahl digestion and the NH-N was determined calorimetrically on an auto analyzer^24^.

To measure the different chemical properties (Ca, K, Na, P, Fe, Mn, Zn), approximately 0.5 g of samples were used for each characteristic. Acid digestion of the sample in digestion tubes was performed using a hot plate to control the temperature for determining the metal by spectroscopic methods using the Thermos Scientific iCE 3300 Atomic Absorption Spectrometer (German) and using 7 ml of H_2_SO_4_ and 2 ml of H_2_O_2_ detectors. The sample was weighed in the digested tube, and then acids were added drop by drop on the inner wall of the tube. The solution was gently stirred to ensure that the sample was harmonized with the acids. The tube was placed on the hot plate until the sample became a clear solution and left to cool until it reached room temperature. Then the solution was transferred to a specific flask, filtered, and the measurement began^25,26^.

Changes in microbial populations during composting were assessed by counting total bacteria, fungi, actinomycetes, cellulolytic bacteria, and proteolytic bacteria. Pathogenic bacterial contaminants, such as *Escherichia coli* and Salmonella, were also enumerated. Culture media used for microbial enumeration were: nutrient agar (NA; Oxoid) for total bacteria, potato dextrose agar (PDA; BD Difco™) for total fungi, starch-casein agar (SCA; HI Media) for total actinomycetes, cellulose agar (CA) for total cellulolytic bacteria, skim milk agar (SMA; Sigma-Aldrich) for total proteolytic bacteria, fecal membrane coliform agar (m-FC; BD Difco™) for total coliform bacteria, and xylose-lysine deoxycholate agar (XLD; Oxoid) for total Salmonella. The spread plate technique was used to estimate the microbial count in each sample (serially diluted) following the methods described by Ravindran et al.^27^. Microbial analysis was performed for all samples in triplicate and expressed as colony-forming units per gram of dry sample.

### Carbon dioxide (CO_2_) and methane (CH_4_) measurements

Greenhouse gas emissions, specifically carbon dioxide (CO₂) and methane (CH₄), were monitored using the closed chamber method, a commonly used method in composting studies due to its reliability in capturing surface gas fluxes. A fixed, sealed chamber was placed above the compost pile at the designated sampling points to trap the emitted gases (using gas storage bags). A flexible plastic tube was connected to the chamber outlet, while the other end was connected to a gas analyzer to ensure consistent, contamination-free sampling.

Gas measurements were performed every 24 h throughout the composting period. During each sampling, gas was drawn from the upper chamber using the tube and analyzed directly using a Geotech GEM5000 portable gas analyzer. This device is capable of accurately detecting and quantifying concentrations of CH_4_, CO_2_, and O_2_ over a wide measurement range (0-100% for each gas), making it suitable for dynamic composting environments. The analyzer was regularly calibrated according to the manufacturer’s guidelines to ensure data accuracy. Cumulative emissions were then calculated using Eq. (3).

3 $${\text{At}} = \left[ {\left( {{\text{t}}_{{\text{b}}} - {\text{t}}_{{\text{a}}} } \right) \times \left( {{\text{F}}_{{{\text{ta}}}} + {\text{F}}_{{{\text{tb}}}} } \right)} \right]/{2}$$

where A_t_ is the cumulative emission between the measurement, days; t_a_ and t_b_ are the dates of ‎measurement, day; and F_ta_ and F_tb_ are the two measurement dates’ gas flows.‎.

### Statistical analysis

All data were tested for homogeneity of variance and the experimental results for maximum temperature obtained, methane and carbon dioxide emissions, and chemical and physical properties of the compost were analyzed using IBM SPSS.25 statistics. Univariate analysis of variance and Duncan’s tests were used to examine the significance of coefficients at *P* = 0.05.

## Results and discussion

### Temperature during composting

Temperature is an important and critical criterion for assessing compost maturity and is an important indicator that reflects the extent of microbial activity during the production process. Therefore, temperatures were constantly recorded throughout the experiment period and were as follows.

**Fig. 4:**
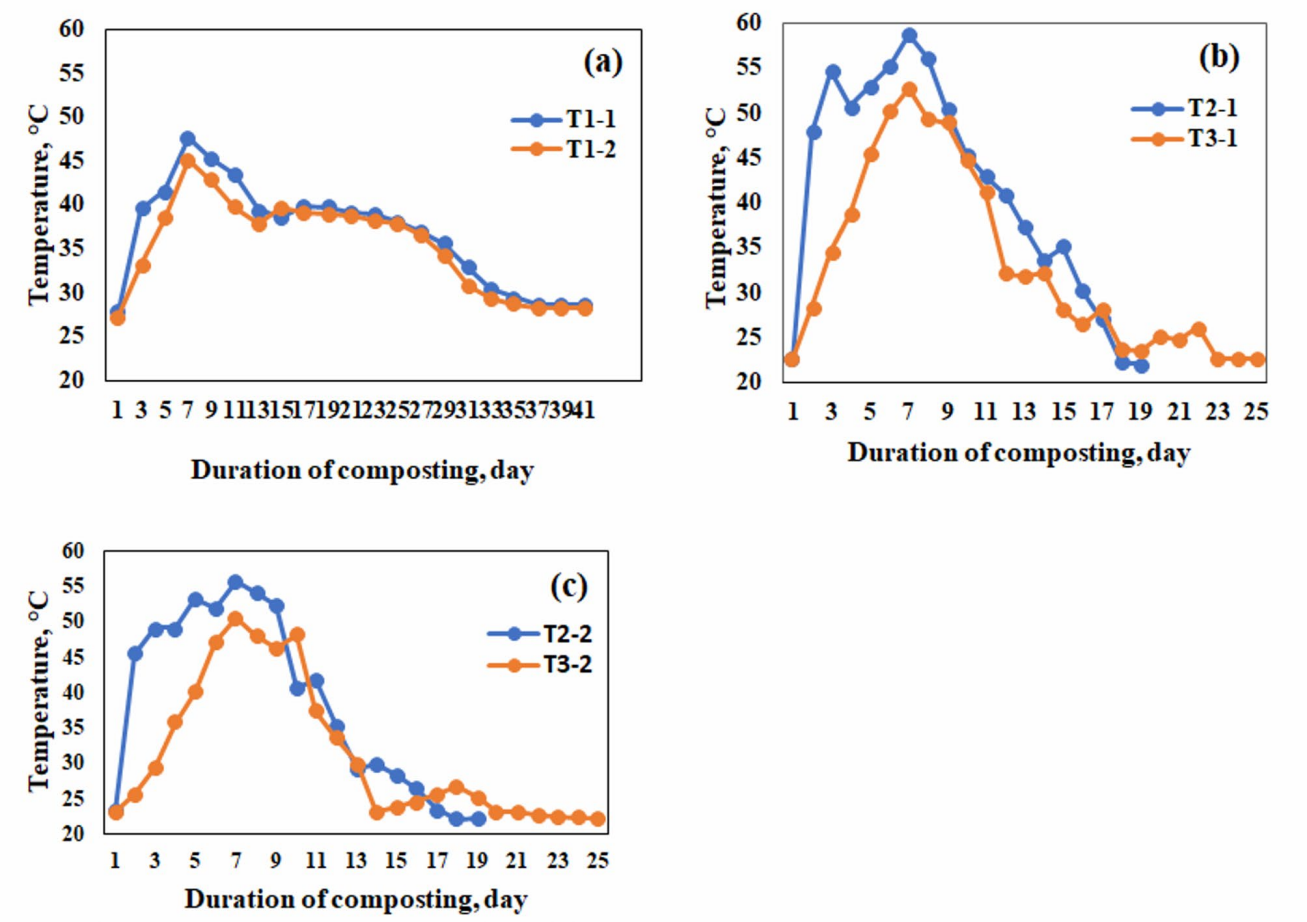
Temperatures changes during the composting process before adding biochar (**a**) control treatment, (**b**) 50:50 treatment, (**c**) 60:40 treatment.

In the first experiment, after adding used oil and inoculum, the temperature reached the maximum in each treatment, with differences between the treatments. The temperature of treatments T_2–1_ and T_2–2_ (50% manure: 50% agricultural residues, and 60% manure: 40% agricultural residues with the addition of 20 ml used cooking oil) reached the thermophilic phase (> 50 °C) on days 2, 3 and continued for 6 days in treatment T_2–1_ (50:50), reaching its highest temperature of 58.74 °C on the 7th day of the experiment (Fig. 4b).

Treatment T_2–2_ (60:40) recorded the highest temperature of 55.7 °C on the 7th day. The thermophilic phase continued for 5 days (Fig. 4c), then the temperature began to drop gradually until the end of the experiment. All thermophilic microorganisms were enhanced and became more active, while all the pathogens could be completely inhibited and the composting process occurred in an ideal way and directed towards maturation. This is consistent with previous studies^28,29^.

The compost matured in T_2–1_ and T_2–2_ treatments after 18 days and reached its final form compared to the remaining treatments which took 25 and 40 days. This was a result of the adaptation of microorganisms that convert compost faster due to the cooking oil used as nutrients compared to other treatments.

Treatments T_3–1_ and T_3–2_ (50% manure: 50% agricultural residues, and 60% manure: 40% agricultural residues + 20% inoculum compost) reached the thermophilic stage (> 50 °C) on the 7th day. The temperature remained above 45 °C in treatment T_3–1_ for 5 days, reaching its peak at 52.7 °C on the 6th day.

Treatment T_3–2_ reached its highest temperature of 50.5 °C on the 7th day. The temperature gradually decreased until the compost matured 25 days after the start of the study, compared to the control treatment which took 40 days (Fig. 4a).

The temperature initially increased due to the rapid degradation of easily biodegradable organic matter, as microorganisms used organic substances for energy and growth. The continuous addition of used oil led to a reduction in volatile solids, as microorganisms preferred the oil over other carbon sources in the mixture. The longer the period of high temperature is the greater the decrease in total volatile solids. According to previous studies Tuzel et al.^30^, have shown that under high temperatures, the degradation of fat, proteins and complex carbohydrates is accelerated, leading to more bioavailable carbon. This explains the higher temperatures resulting from the addition of used oil. The addition of used oil and compost as inoculum increased the temperature by 23.5% and 11.9% over the control treatment, respectively, reducing the compost maturity period from 40 days to 18 and 25 days respectively.

**Fig. 5:**
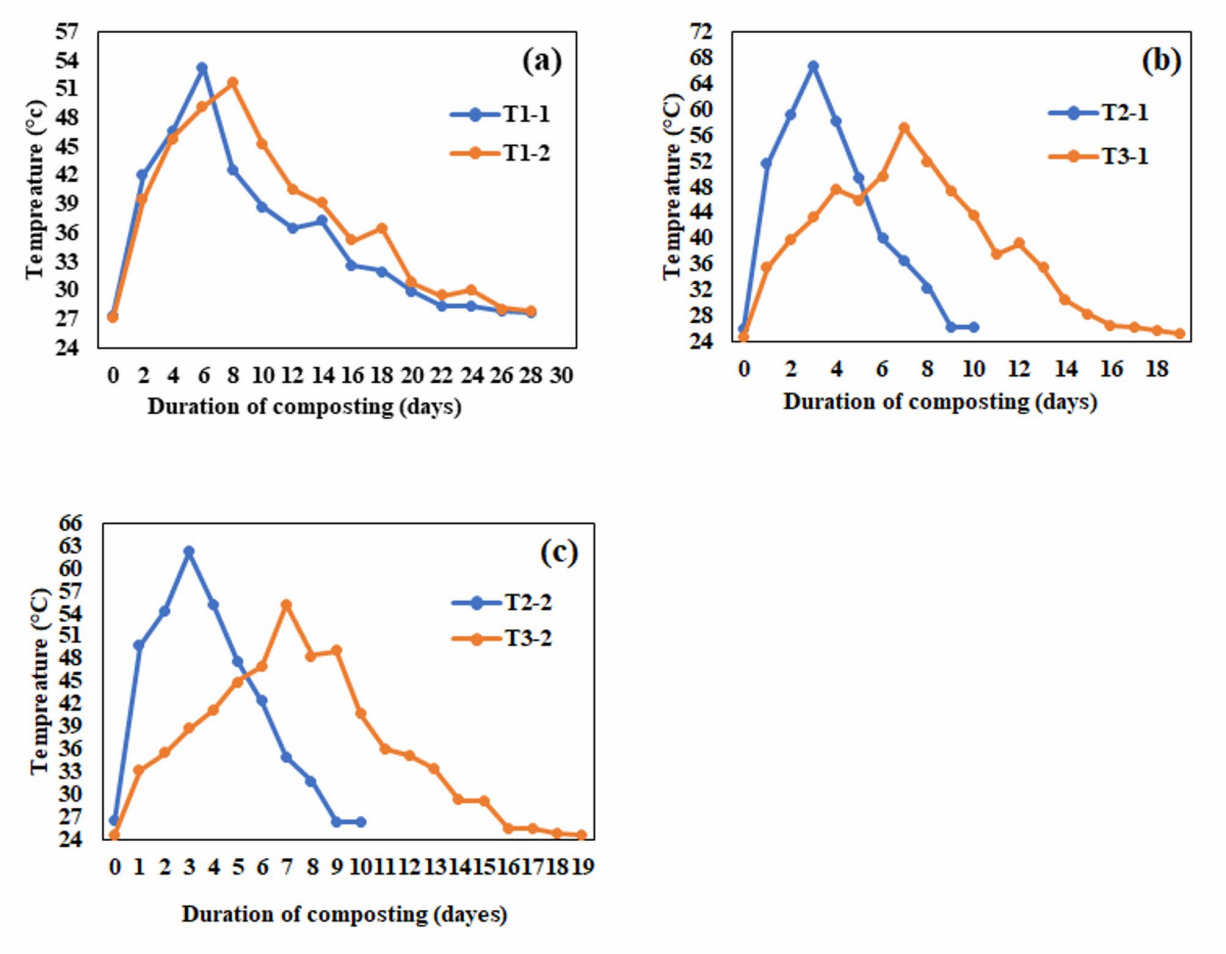
Temperatures evaluation during the composting process after adding biochar (**a**) control treatment, (**b**) 50:50 treatment, (c) 60:40 treatment.

After adding biochar in second experiment, the temperature of the treatments increased by approximately 5–6 °C. In all treatments, there was a rapid rise in the compost temperature, and in some treatments, the thermophilic stage was reached from the first day. The first treatment (T_1–1_, T_1–2_) reached its highest temperatures of 53.2 °C and 51.6 °C on the 6th and 8th day of the experiment, and the compost matured in this treatment after 30 days (Fig. 5a).

The second treatment (T_2–1_, T_2–2_) recorded the highest temperatures compared to the rest of the treatments, reaching the thermophilic stage at the end of the first day and reaching its maximum temperature of 66.6 °C and 62.2 °C on the 4th and 5th day from the beginning of the experiment. Due to the high temperatures, the ripening period decreased, and the compost matured only 10 days after the start of the experiment (Fig. 5b).

**Fig. 6:**
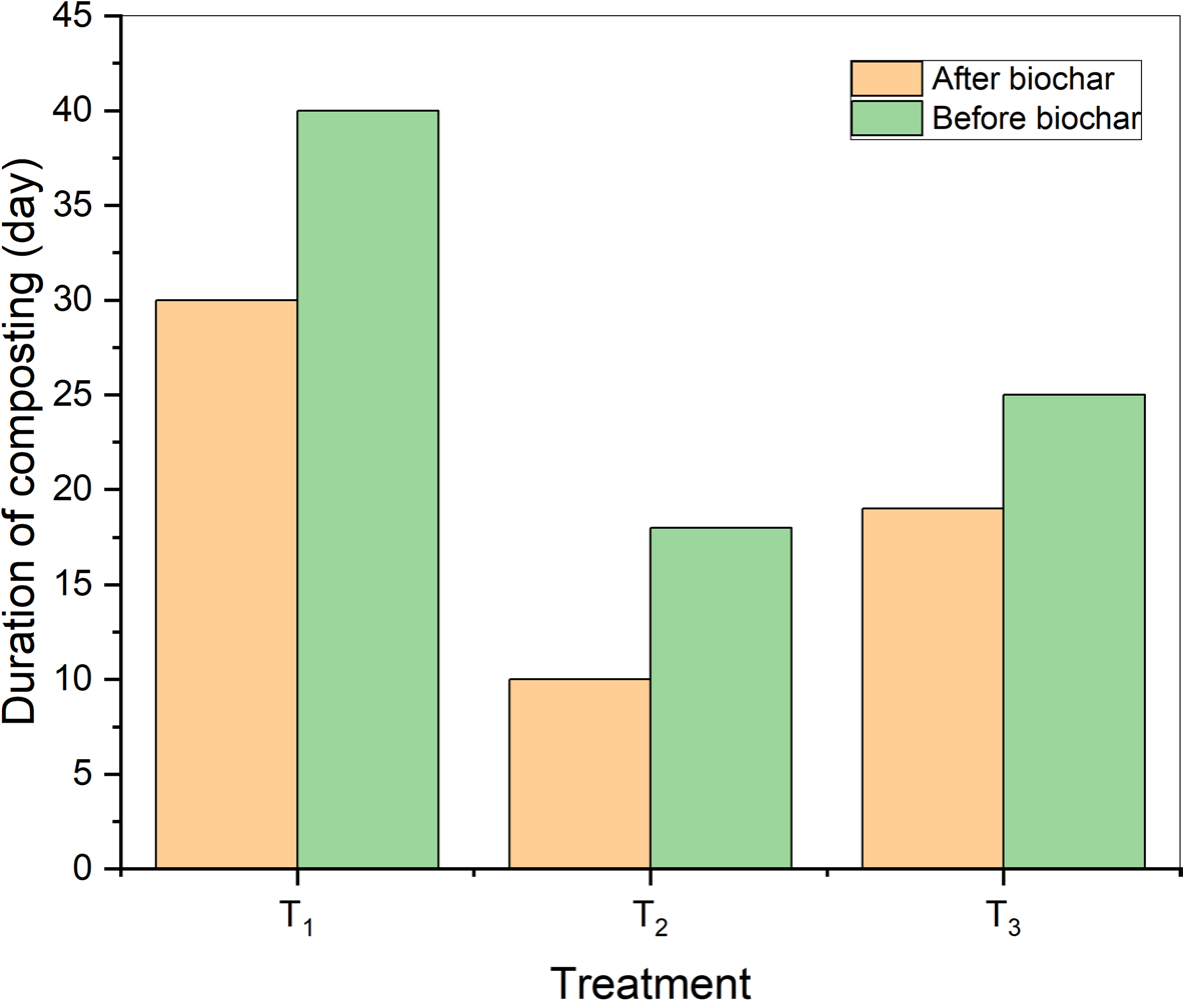
Duration of the composting process during treatments before and after adding biochar.

The third treatment (T_3–1_, T_3–2_) reached its highest temperature of 57.3 °C and 55.1 °C on day 7 of the experiment, and compost maturity was completed after 19 days (Fig. 5c). The addition of biochar increased microbial respiration and raised temperatures, accelerating the compost ripening process and reducing its duration. Compared to the results of the second experiment, the maturity of compost was reduced in the first treatment from 40 to 30 days, in the second treatment from 18 to 10 days, and in the third treatment from 25 to 19 days, as shown in Fig. 6. This is attributed to the fact that biochar has a large surface area and high absorption capacity, which enhances the spread of microbes, as mentioned in previous studies^1,31^. There were clear significant differences between all treatments, with treatment (T_1−2_) showing the least significant difference (a) and treatment (T_2−1_) showing the highest significant difference (f) as shown in Table 4.

**Table 4:** The standard deviation (*p* < 0.05) of the mean value between the different treatments for maximum temperature before and after adding biochar.

Treatment	Before adding biochar			After adding biochar		
	Mean	Std. deviation	Sig*.	Mean	Std. deviation	Sig*.
T_1–1_	47.7	0.656	b	53.2	0.436	b
T_1–2_	45.1	0.173	a	51.6	0.211	a
T_2–1_	58.74	0.175	f	66.8	0.322	f
T_2–2_	55.7	0.361	e	62.2	0.216	e
T_3–1_	52.7	0.458	d	57.3	0.361	d
T_3–2_	50.5	0.436	c	55.1	0.173	c

*Values with different letters indicate significant (*p* < 0.05).

T_1–1_:50% manure + 50% agriculture residues, T_1–2_: 60% manure + 40% agriculture residues, T_2–1_: 50% manure + 50% agriculture residues + 20 ml used cooking oil, T_2–2_: 60% manure + 40% agriculture residues + 20 ml used cooking oil, T_3–1_: 50% manure + 50% agriculture residues + 20% inoculum compost, T_3–2_: 60% manure + 40% agriculture residues + 20% inoculum compost.

### Greenhouse gas emissions during the composting process

#### Carbon dioxide (CO_2_)

CO_2_ emissions increased at the beginning of the composting process due to the rapid degradation of easily biodegradable organic materials. CO_2_ emissions serve as a key indicator of organic matter degradation and increased microbial activity, in line with findings by^2,32^.Variations in CO_2_ emissions correlated with temperature a cross all treatments, with emissions positively linked to maximum temperatures. Generally, CO_2_ emissions per treatment were highest at the start of the thermophilic stage when organic material degradation was most rapid, gradually decreasing towards to the end, consistent with Dume et al.^33^ and Feng et al.,^34^.

**Fig. 7:**
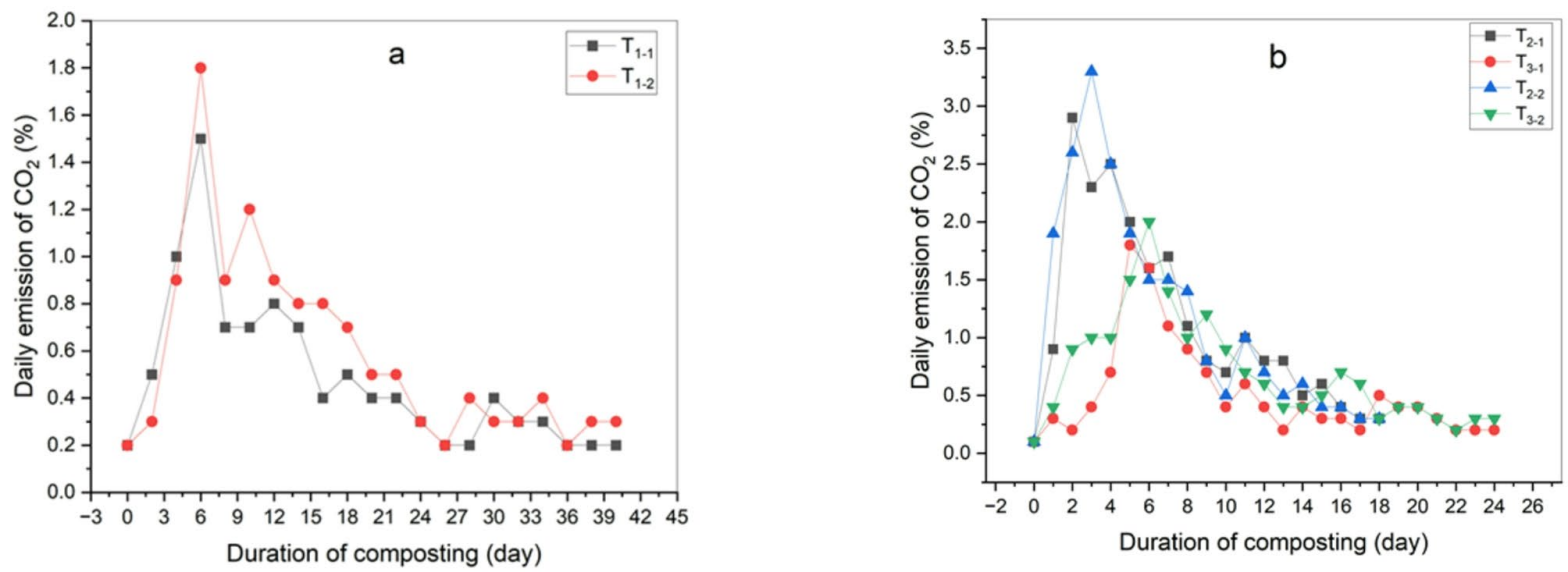
Daily emission of CO_2_ during the composting process before adding biochar (**a**) control treatment, (**b**) 50:50 treatment and 60:40 treatment.

**Fig. 8:**
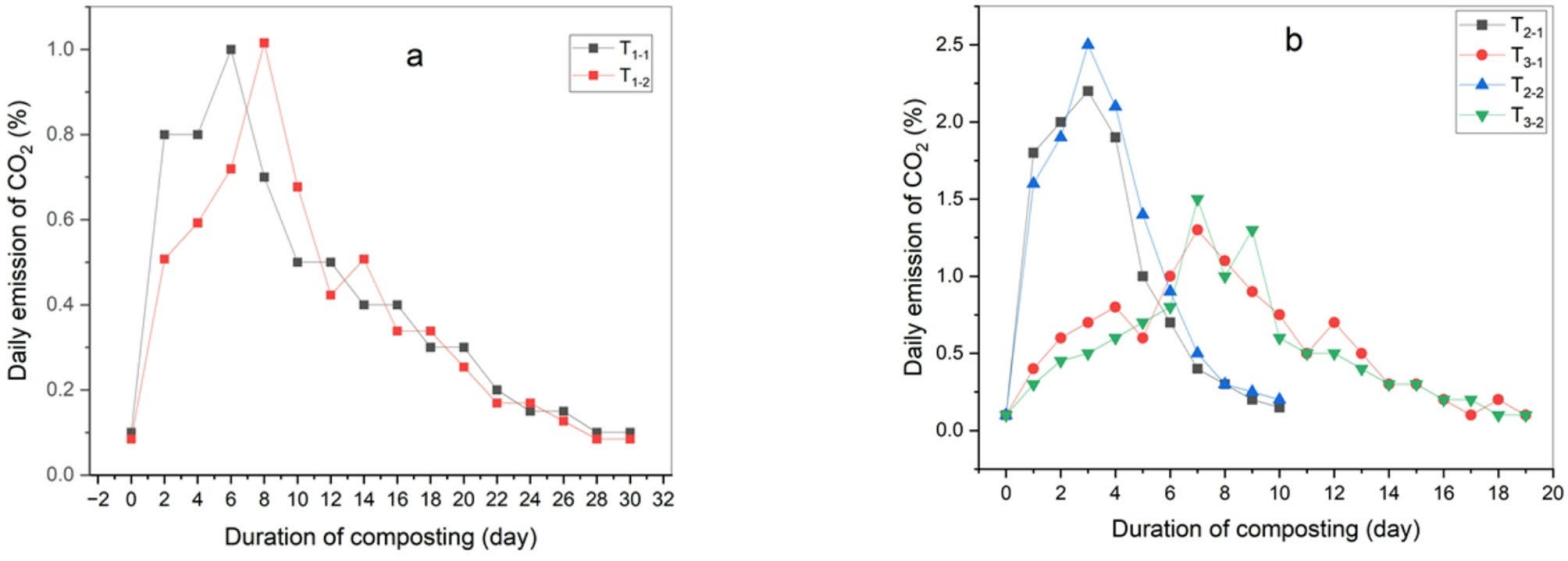
Daily emission of CO_2_ during the composting process after adding biochar (**a**) control treatment, (**b**) 50:50 treatment and 60:40 treatment.

In this study, the treatment with used oil exhibited the highest daily CO_2_ emissions compared to other treatments, attributed to the high temperatures promoting accelerated decomposition process. However, the total emissions over the experiment period were lower due to the shorter compost maturation period ‎compared to other treatments. The results showed that the maximum CO_2_ emissions were in treatment T_2–2_ (3.3%), followed by treatments T_2–1_ and T_3–2_ (2.9%, 2%), then T_3–1_, T_1–2_, T_1–1_ (1.8%, 1.8%, 1.5%) as shown in Fig. 7. After adding biochar, CO_2_ emissions increased in the first days, especially in the thermophilic stage, which is proportional to the temperature. By adding biochar, the temperature rose quickly and thus CO_2_ emissions increased due to its properties that improved the way oxygen is distributed inside the compost. Then the CO_2_ emissions gradually decreased until the end of the maturity period of the compost evidence of the degradation of organic matter and the absence of an anaerobic environment as shown in Fig. 8.

By comparing CO_2_ emissions before and after adding biochar in this experiment, it was observed that these emissions decreased at rates ranging between 24 and 33%, with the highest daily emission recorded in treatment T_2−2_ (2.5%), followed by treatments T_2−1_, T_3−2_ (2.2%,1.5%), then T_3−1_, T_1–2_, T_1−1_ (1.3%,1.2%,1.1%). This is probably due to biochar’s high absorption capacity and its ability to exchange cationic and larger pores, which greatly enhances the production process of the compost and the reproduction of microorganisms. This is consistent with what has been stated by Cho et al.^35^. These results are also similar to the study conducted by Geng et al.^36^, which demonstrated that biochar can effectively reduce greenhouse gas emissions during composting by changing the production environment and regulating the microbial community environment. There were clear significant differences between all treatments before adding biochar, with treatment (T_1−1_) showing the least significant difference (a) and treatment (T_2−2_) showing the highest significant difference (f). After adding biochar treatment (T_1−1_) showed the least significant difference (a) and treatment (T_3−1_) showed the highest significant difference (df) as shown in Table 5.

**Table 5:** The standard deviation (*p* < 0.05) of the mean value between the different treatments for total emission of CO_2_ during the composting before and after adding biochar.

Treatment	Before adding biochar			After adding biochar		
	Mean	Std. deviation	Sig*.	Mean	Std. deviation	Sig*.
T_1–1_	10.1	0.312	a	6.30	0.367	a
T_1–2_	12	0.274	b	7.1	0.240	b
T_2–1_	21.3	0.635	e	10.75	0.346	cd
T_2–2_	22.2	0.284	f	11.75	0.377	e
T_3–1_	12.8	0.541	c	11.12	0.382	df
T_3–2_	17.5	0.361	d	10.47	0.342	c

*Values with different letters indicate significant (*p* < 0.05).

T_1–1_:50% manure + 50% agriculture residues, T_1–2_: 60%manure + 40% agriculture residues, T_2–1_: 50% manure + 50% agriculture residues + 20 ml used cooking oil, T_2–2_: 60% manure + 40% agriculture residues + 20 ml used cooking oil, T_3–1_: 50% manure + 50% agriculture residues + 20% inoculum compost, T_3–2_: 60% manure + 40% agriculture residues + 20% inoculum compost.

#### Methane (CH_4_)

Methane contributes significantly to global warming and is one of the main greenhouse gases produced during the composting process^37^. Methane is produced under the influence of methanogens in an anaerobic environment, where its production is due to the deoxidation of methanogen from CO_2_/H_2_ and acetic acid in situations with low oxygen levels according to Wang et al.^38^ and Yang et al.^39^.

**Fig. 9:**
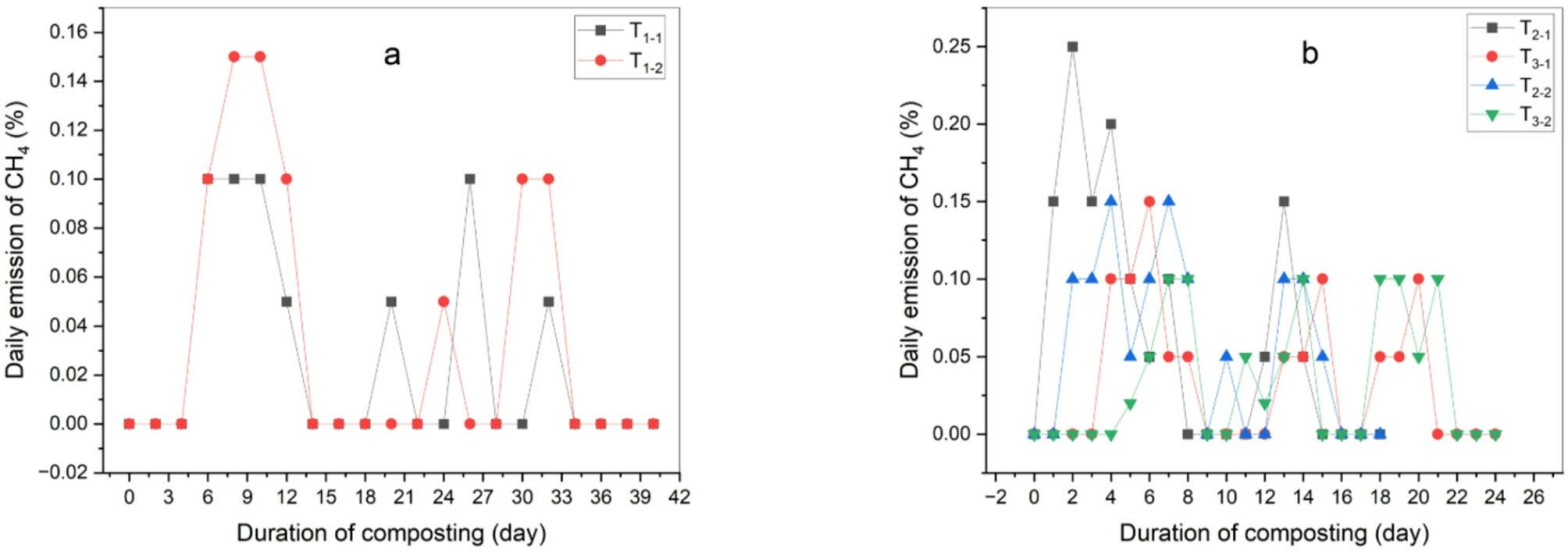
Daily emission of CH_4_ during the composting process before adding biochar (**a**) control treatment, (**b**) 50:50 treatment and 60:40 treatment.

In this experiment, methane concentrations in all treatments reached their maximum at the beginning of the composting process, as shown in Fig. 9, which is consistent with studies that discovered that the highest level of methane emission occurs at the beginning of the compost process^40,41^. The first treatment (T_1_) recorded the highest daily emission of CH_4_ at 0.15%, as shown in Fig. 11a, while the second treatment (T_2_) after adding used cooking oil reached 0.25% and the third treatment (T_3_) recorded the highest daily emission of 0.14% as shown in Fig. 9b. There was a positive correlation between high temperatures and methane emissions, as methane emissions were mostly found in the thermophilic stage. This is because microorganisms can quickly decompose organic materials at this stage, leading to a decrease in oxygen levels which agree with Ma et al.^42^, Wang et al.^43^ and Yang et al.^32^.

With the degradation of organic matter and oxygen regeneration, methane emissions decreased significantly and remained low until the end of the experiment as found by Yang et al.^32^.

**Fig. 10:**
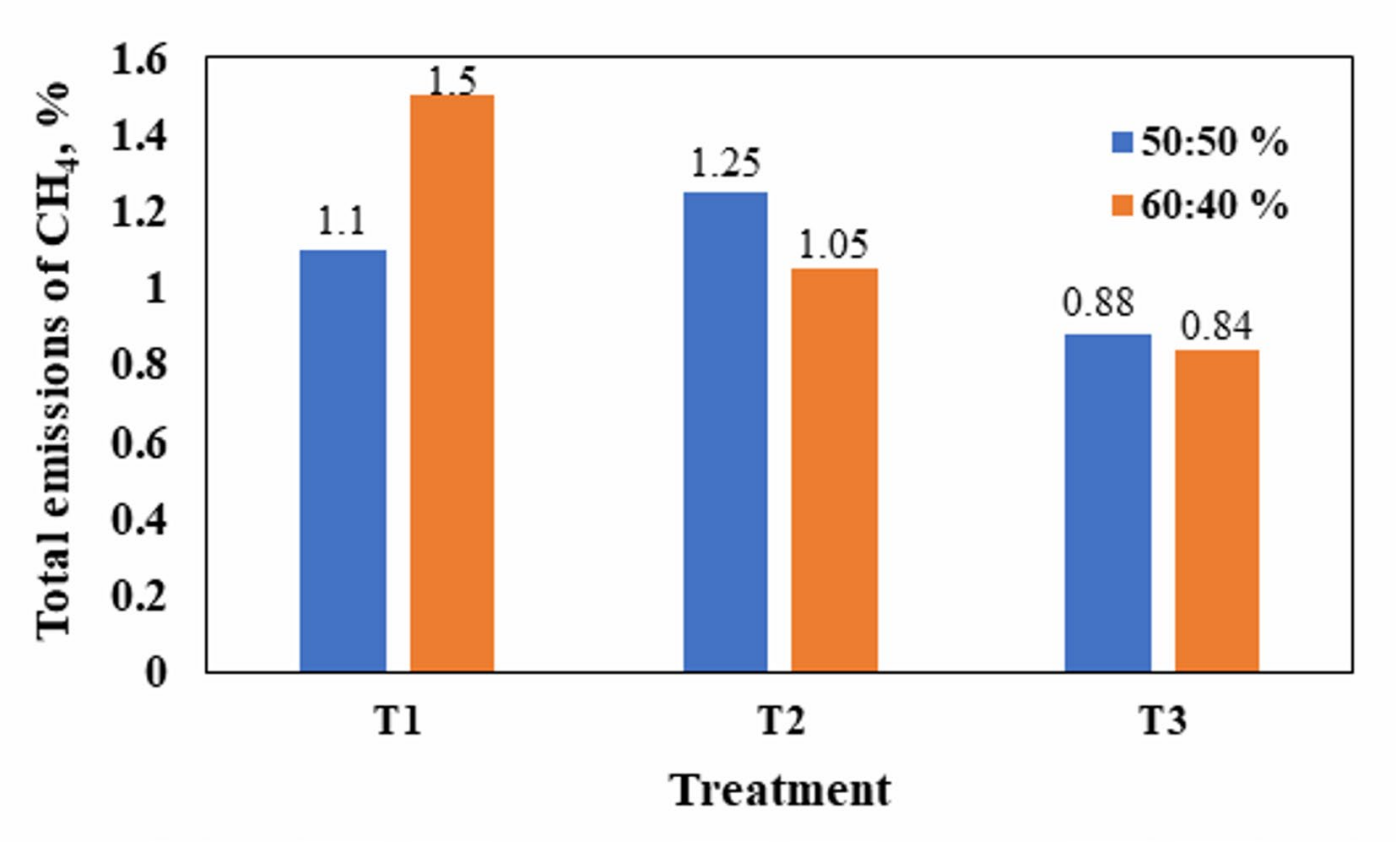
Total emission of CH_4_ during the composting process.

**Fig. 11:**
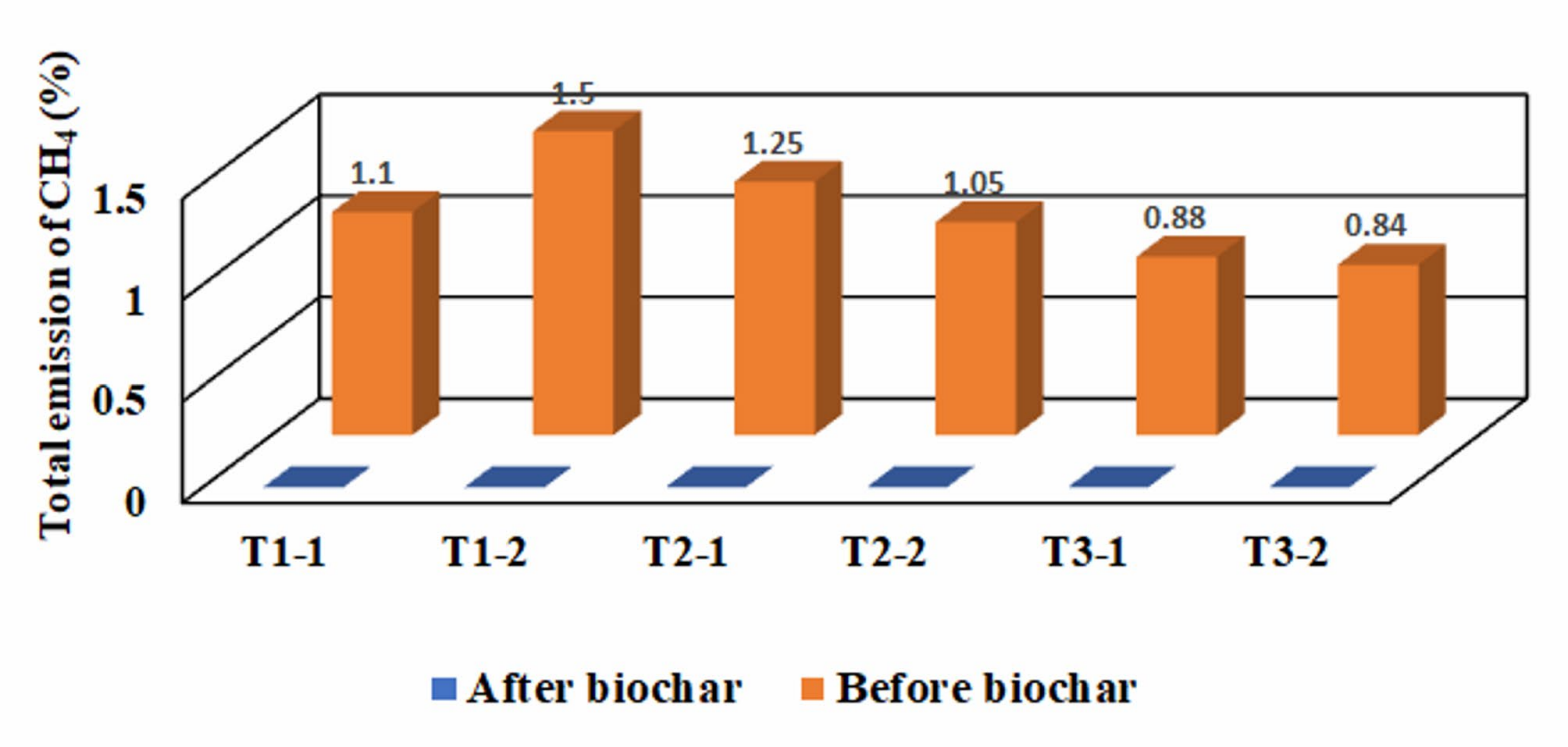
Total emission of CH_4_ during the composting process before and after adding biochar. “CH_4_ = 0” indicates values below the detection limit.

In this experiment, methane emissions reached zero in many cases due to control factors implemented during the maturity period, such as ventilation and moisture control. Additionally, the reduction in the number of days of compost maturity in treatments 2 (adding used cooking oil) and 3 (adding inoculum compost) led to a decrease in CO_2_ and CH_4_ emissions, contributing to reducing global warming. Although treatment 2 (adding used cooking oil) produced the highest daily emissions of CH_4_ due to reduced maturity, its cumulative methane emissions were not greater than other treatments (Fig. 10). There were no significant differences between treatments, with treatments (T_2–2_, T_3–1_, T_3–2_) showing the same difference (a). However, there was an increase in significant differences for the remaining treatments as shown in Table 6. After adding biochar, no methane emissions were recorded throughout the experiment period in all treatments, as the device’s reading indicated zero methane emissions per day in all treatments (Fig. 11). This may have occurred due to the porous properties of biochar, which improved the distribution of oxygen within the compost, creating an anaerobic environment for methane production in addition to the control factors controlled throughout the experiment.

**Table 6:** The standard deviation (*p* < 0.05) of the mean value between the different treatments for total emission of CH_4_ during the composting before adding biochar.

Treatment	Mean	Std. deviation	Sig*.
T_1–1_	1.1	0.265	ab
T_1–2_	1.5	0.284	b
T_2–1_	1.25	0.2	ab
T_2–2_	1.05	0.1	a
T_3–1_	0.88	0.267	a
T_3–2_	0.84	0.144	a

*Values with different letters indicate significant (*p* < 0.05).

T_1–1_:50% manure + 50% agriculture residues, T_1–2_: 60% manure + 40% agriculture residues, T_2–1_: 50% manure + 50% agriculture residues + 20 ml used cooking oil, T_2–2_: 60% manure + 40% agriculture residues + 20 ml used cooking oil, T_3–1_: 50% manure + 50% agriculture residues + 20% inoculum compost, T_3–2_: 60%manure + 40% agriculture residues + 20% inoculum compost.

### Chemical characteristics of compost and Microbiological

#### pH

**Fig. 12:**
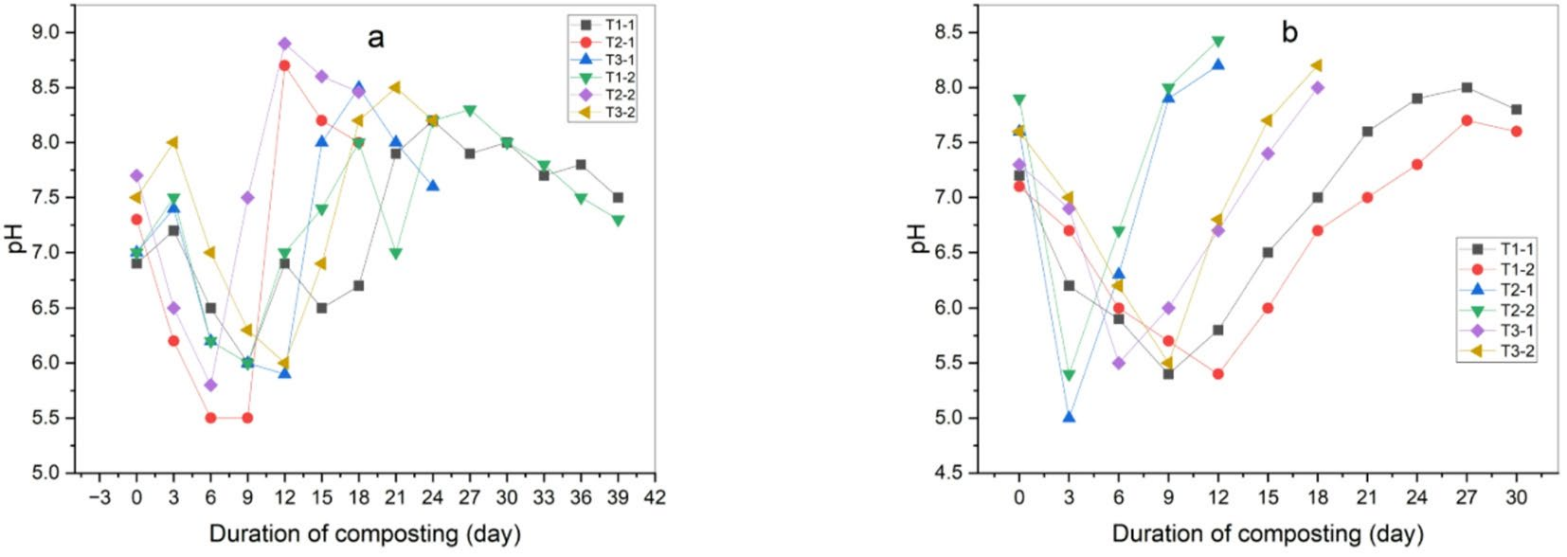
pH of composting process (**a**) before adding biochar, (**b**) after adding biochar.

During the initial phase of the composting process there can be a decrease in pH values as a result of microorganisms’ activities breaking down organic materials and producing both organic and inorganic acids. This is indicated by the results of changes in pH values in this experiment in (Fig. 12a), which agrees with Koura et al.^29^. The microbial activity that produces carbon dioxide (CO_2_) and increases the concentration of organic acids may also be the cause of the pH values dropping. During successful compost processing and as a result of good ventilation, acids degrade rapidly and are themselves used by the microbial community leading to a rise in pH values which agree with Zahrim et al.^44^.

High pH values can also be explained due to the organic matter’s mineralization and ammonia production through microbial activities which agrees with Hwang et al.^45^. During the thermophilic phase and as a result of high temperature the pH remains in the weak alkaline range or acid and the possibility of this occurrence is because of the nitrogen lost as a result of emissions of ammonia (NH_3_), as confirmed by^46,47^. But at the end of the compost process, as a result of the microbial nitrification process, the ammonia nitrogen volatilizes and hydrogen is released, so the pH values slow down. After adding biochar, the pH values of the compost were enhanced, as the pH values increased at the end of the compost production stage, but variations appeared during the thermophilic stage before and after adding biochar, as the pH values were low at this stage after adding biochar as shown in Fig. 12b.

High pH values after adding biochar are due to the composition of organic acids, nitrogen ammonia and also as a result of the effect of adding biochar on carbon dioxide emissions, as the decrease in carbon dioxide emissions led to an increase in the pH values of the compost. Treatments showed significant differences between them but not as large, where treatment (T_1–2_) showed the least significant difference (a) and treatment (T_2–2_) showed the highest significant difference (c) as shown in Table 7.

**Table 7:** The standard deviation (*p* < 0.05) of the mean value between the different treatments for final pH before and after adding biochar.

Treatment	Before adding biochar			After adding biochar		
	Mean	Std. deviation	Sig*.	Mean	Std. deviation	Sig*.
T_1–1_	7.5	0.361	ab	7.8	0.526	ab
T_1–2_	7.3	0.469	a	7.6	0.291	a
T_2–1_	8	0.362	bc	8.2	0.374	ab
T_2–2_	8.46	0.235	c	8.43	0.467	b
T_3–1_	7.6	0.356	ab	8.2	0.308	ab
T_3–2_	7.9	0.223	abc	8.2	0.377	ab

*Values with different letters indicate significant (*p* < 0.05).

T_1–1_:50% manure + 50% agriculture residues, T_1–2_: 60% manure + 40% agriculture residues, T_2–1_: 50% manure + 50% agriculture residues + 20 ml used cooking oil, T_2–2_: 60%manure + 40% agriculture residues + 20 ml used cooking oil, T_3–1_: 50% manure + 50% agriculture residues + 20% inoculum compost, T_3–2_: 60% manure + 40% agriculture residues + 20% inoculum compost.

#### Electrical conductivity (EC)

**Fig. 13:**
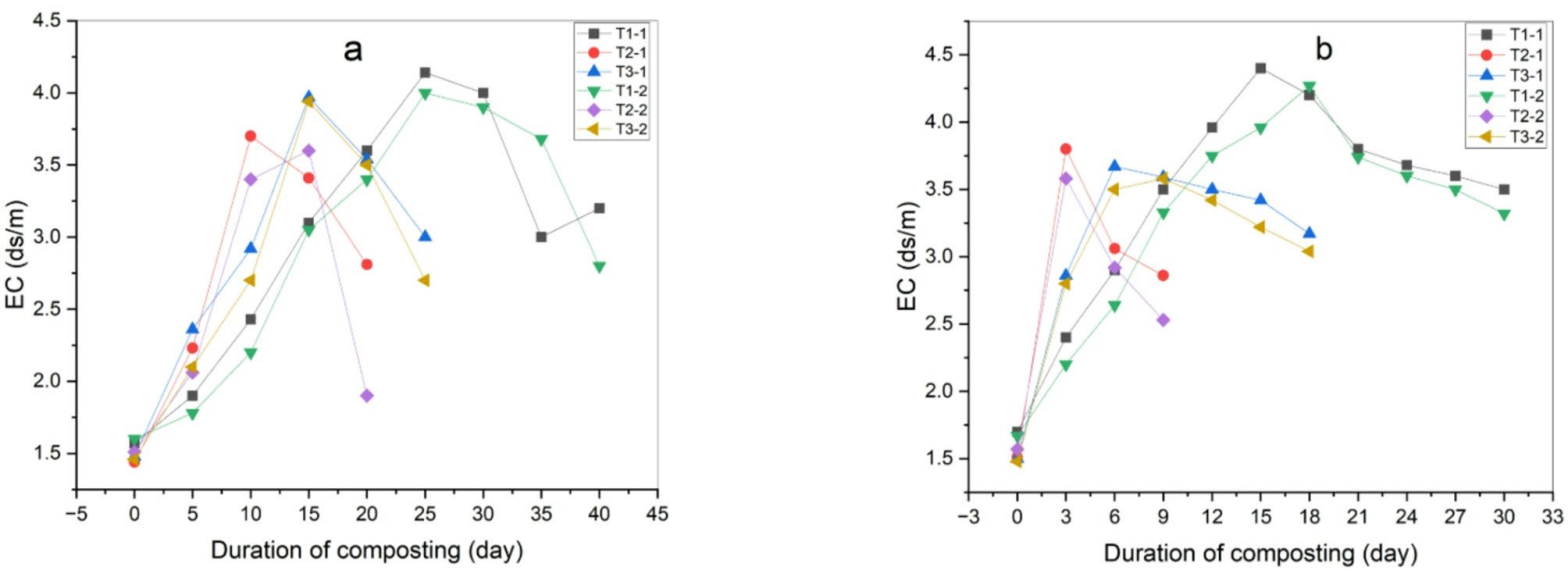
EC of composting process (**a**) before adding biochar, (**b**) after adding biochar.

Figure (13a) shows the changes in electrical conductivity (EC) for all treatments. This data indicates that the EC in the first phase of the composting process increased in all treatments. This can be explained by the fact that mineral ions including phosphates, ammonium, potassium, and other cations are released during the breakdown of organic matter. Then values gradually decreased until the end due to ammonia volatilization and mineral salts deposition. This is in line with the findings of research conducted by Ramnarain et al. and Zahrim et al.^44,48^ that showed increased electrical conductivity as a result of the breakdown of organic materials and the release of minerals like P, K, Mg, Ca, which are interchangeable in available formats on cations image. Initially, after adding biochar, with the rapid increase in temperature and improvement of aeration and porosity of the compost, this led to the creation of a more suitable environment for the spread of microorganisms, thus accelerating the decomposition of organic materials and increasing the concentration of soluble nutrients, causing a rapid increase in electrical conductivity (Fig. 13b). However, as the compost process progressed and the compost ripening period was accelerated as a result of the addition of biochar, the electrical conductivity decreased due to the biochar absorbing large amounts of salt ions because of its structural properties. Additionally, biochar is often alkaline, causing the availability of inorganic nutrients and thus a decrease in electrical conductivity, which is agreed with Chaher et al.^49^. There were significant differences between all treatments before adding biochar, where treatment (T_2–2_) showed the least significant difference (a) and treatment (T_1–1_) showed the highest significant difference (c). While there were no significant differences between treatments after adding biochar as shown in Table 8.

**Table 8:** The standard deviation (*p* < 0.05) of the mean value between the different treatments for final EC before and after adding biochar.

Treatment	Before adding biochar			After adding biochar		
	Mean	Std. deviation	Sig*.	Mean	Std. deviation	Sig.
T_1–1_	3.9	0.387	c	3.5	0.755	a
T_1–2_	3.6	0.280	bc	3.32	0.546	a
T_2–1_	3.2	0.269	ab	2.86	0.318	a
T_2–2_	2.8	0.410	a	2.53	0.632	a
T_3–1_	3.5	0.211	bc	3.17	0.651	a
T_3–2_	3.3	0.354	abc	3.04	0.413	a

*Values with different letters indicate significant (*p* < 0.05).

T_1–1_:50% manure + 50% agriculture residues, T_1–2_: 60% manure + 40% agriculture residues, T_2–1_: 50% manure + 50% agriculture residues + 20 ml used cooking oil, T_2–2_: 60% manure + 40% agriculture residues + 20 ml used cooking oil, T_3–1_: 50% manure + 50% agriculture residues + 20% inoculum compost, T_3–2_: 60% manure + 40% agriculture residues + 20% inoculum compost.

#### The C:N ratio, total carbon (TC) and total nitrogen (TN)

The initial C:N ratio is considered a crucial and significant component in ensuring the success of the composting process. The ideal proportion is between 25 and 35%, so it was calculated before the experiments started and set at 30%. Deviation from this ratio inhibits microbial activity due to insufficient carbon sources available and causes the raw materials to deteriorate slowly and incompletely. Conversely, an increase indicates inadequate nitrogen supply, which limits microbial metabolism. As time passes and the composting process progresses, the C:N ratio decreases due to the mineralization of substrates or an increase in total nitrogen concentration after the decomposition of carbon and the deterioration of nutrient material until the end of the composting process as mentioned by Esmaeili et al.^50^. The addition of biochar had a limited effect on the C:N ratio because in biochar, the majority of carbon is stable, however, led to an increase in the nitrogen ratio by reducing the emission of volatile ammonia, thus decreasing nitrogen loss. Additionally, due to its high porosity and specific surface area, biochar accelerated the breakdown of organic matter, enhancing and enriching total nitrogen. These results are similar to the study conducted by Awasthi et al.^13^, where a significant reduction in total carbon and nitrogen losses was observed with the addition of bamboo biochar at different ratios. A C:N ratio of less than 20 is an indicator of maturity, which may be attributed to the decomposition of organic matter as organic carbon deteriorates and total nitrogen increases^51^.

Temperature change, moisture content, pH, C:N ratio, and electrical conductivity are key indicators of the composting process. The final samples were analyzed to evaluate of the characteristics of the composting materials for all treatments. The analysis indicated significant differences in some characteristics and no significant differences in others, as shown in Tables 9, 10. In this study, the addition of used cooking oil increased emissions, leading to higher nitrogen and carbon losses by 13% and 15% compared to the first and third treatments, resulting in an increased C:N ratio. However, after adding biochar, there was a more effective stabilize carbon and nitrogen, reducing nitrogen and carbon losses by 30%, 53% and 39% and significantly decreasing methane emissions.

**Table 9:** Chemical properties of compost before and after adding biochar.

Treatment	Before adding biochar				After adding biochar			
	MC (%)	TC (%)	TN (%)	C: *N* ratio	MC (%)	TC (%)	TN (%)	C: *N* ratio
T_1–1_	37 ± 3 bc	29.6 ± 0.23 a	2.22 ± o.23 b	15.4 ± 1.82a	31 ± 2.3b	35.97 ± 0.66 c	3.21 ± 0.41 a	11.2 ± 2.46 a
T_1–2_	34 ± 1.4 ab	30.1 ± 0.28 a	2.02 ± 0.16 b	15 ± 1.28a	33 ± 1.9 bc	36.35 ± 0.44 c	3.29 ± 0.38 a	11.04 ± 1.21 a
T_2–1_	28 ± 2 a	32.89 ± 0.55 c	1.92 ± 0.26 a	17.1 ± 1.05 b	25 ± 2 a	37.84 ± 0.39 d	4.08 ± 0.62 c	9.3 ± 2.01 a
T_2–2_	30 ± 0.8 a	30.74 ± 0.33 b	1.74 ± 0.25 a	17.7 ± 1.3 b	28 ± 2.6 b	36.34 ± 0.41 c	3.72 ± 0.46 b	9.8 ± 1.38 a
T_3–1_	35 ± 2.1 b	31.12 ± 0.24 b	2.1 ± 0.3 ab	14.8 ± 1.4a	33 ± 1.7 bc	33.98 ± 0.32 b	3.45 ± 0.66 ab	9.85 ± 1.49 a
T_3–2_	40 ± 1.3 cd	30.09 ± 0.19 a	2.19 ± 0.31 ab	13.7 ± 1.8a	35 ± 2.2 c	32.86 ± 0.42 a	3.564 ± 0.33 ab	9.2 ± 0.59 a

T_1–1_:50%manure + 50% agriculture residues, T_1–2_: 60% manure + 40% agriculture residues, T_2–1_: 50% manure + 50% agriculture residues + 20 ml used cooking oil, T_2–2_: 60% manure + 40% agriculture residues + 20 ml used cooking oil, T_3–1_: 50% manure + 50% agriculture residues + 20% inoculum compost, T_3–2_: 60% manure + 40% agriculture residues + 20% inoculum compost. The values indicate mean ± standard error (*n* = 3).

**Table 10:** Characteristics of compost before and after adding biochar (continued).

Treatment	Before adding biochar					After adding biochar				
	Organic matter (%)	K (%)	*P* (%)	pH	EC (ds/m)	Organic matter (%)	K (%)	*P* (%)	pH	EC (ds/m)
T_1–1_	64.91 ± 1.49 d	0.54 ± 0.13 b	0.88 ± 0.25 a	7.5 ± 0.36 ab	3.9 ± 0.39 c	52.8 ± 1.3 d	0.73 ± 0.17 b	1.8 ± 0.35 a	7.8 ± 0.53 ab	3.5 ± 0.76a
T_1–2_	67.4 ± 2.61 d	0.23 ± 0.05 a	0.78 ± 0.27 a	7.3 ± 0.47 a	3.6 ± 0.28 bc	56.1 ± 0.95 e	0.44 ± 0.2 a	1.2 ± 0.18 a	7.6 ± 0.29 a	3.32 ± 0.56 a
T_2–1_	43.73 ± 1.53 b	0.31 ± 0.07 a	0.70 ± 0.13 a	8 ± 0.36 bc	3.2 ± 0.27 ab	38.7 ± 1.82 c	0.63 ± 0.12 b	1.98 ± 0.31 a	8.2 ± 0.37 ab	2.86 ± 0.32 a
T_2–2_	48.93 ± 1.73 c	0.23 ± 0.04a	0.74 ± 0.13 a	8.46 ± 0.24 c	2.8 ± 0.41 a	40.3 ± 0.74 c	0.69 ± 0.27 b	1.4 ± 0.27 a	8.43 ± 0.47 b	2.53 ± 0.63 a
T_3–1_	46.7 ± 2.2 a	0.28 ± 0.08 a	0.86 ± 0.09 a	7.6 ± 0.36 ab	3.5 ± 0.21 bc	37.4 ± 1.6 b	0.54 ± 0.39 a	1.92 ± 0.42 a	8.2 ± 0.32 ab	3.17 ± 0.65 a
T_3–2_	45.2 ± 0.5 a	0.23 ± 0.06 a	0.83 ± 0.12 a	7.9 ± 0.23 abc	3.3 ± 0.35 abc	33.2 ± 2.32 a	0.58 ± 0.22 a	1.6 ± 0.14 a	8.2 ± 0.38 ab	3.04 ± 0.43 a

T_1–1_:50%manure + 50% agriculture residues, T_1–2_: 60% manure + 40% agriculture residues, T_2–1_: 50% manure + 50% agriculture residues + 20 ml used cooking oil, T_2–2_: 60% manure + 40% agriculture residues + 20 ml used cooking oil, T_3–1_: 50% manure + 50% agriculture residues + 20% inoculum compost, T_3–2_: 60% manure + 40% agriculture residues + 20% inoculum compost.

#### Changes in Microbiological composition during composting

**Fig. 14:**
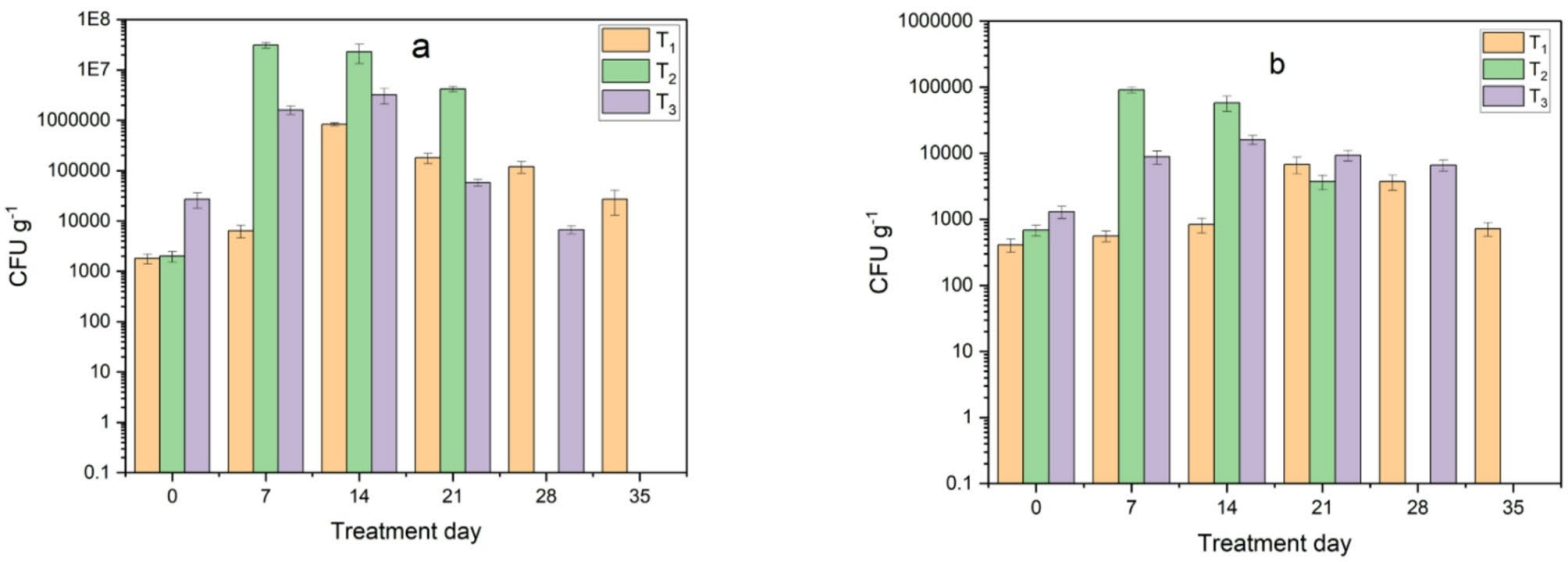
Monitoring of microbial load in treatments during composting before adding biochar: (**a**) total bacteria and (**b**) total fungi.

**Fig. 15:**
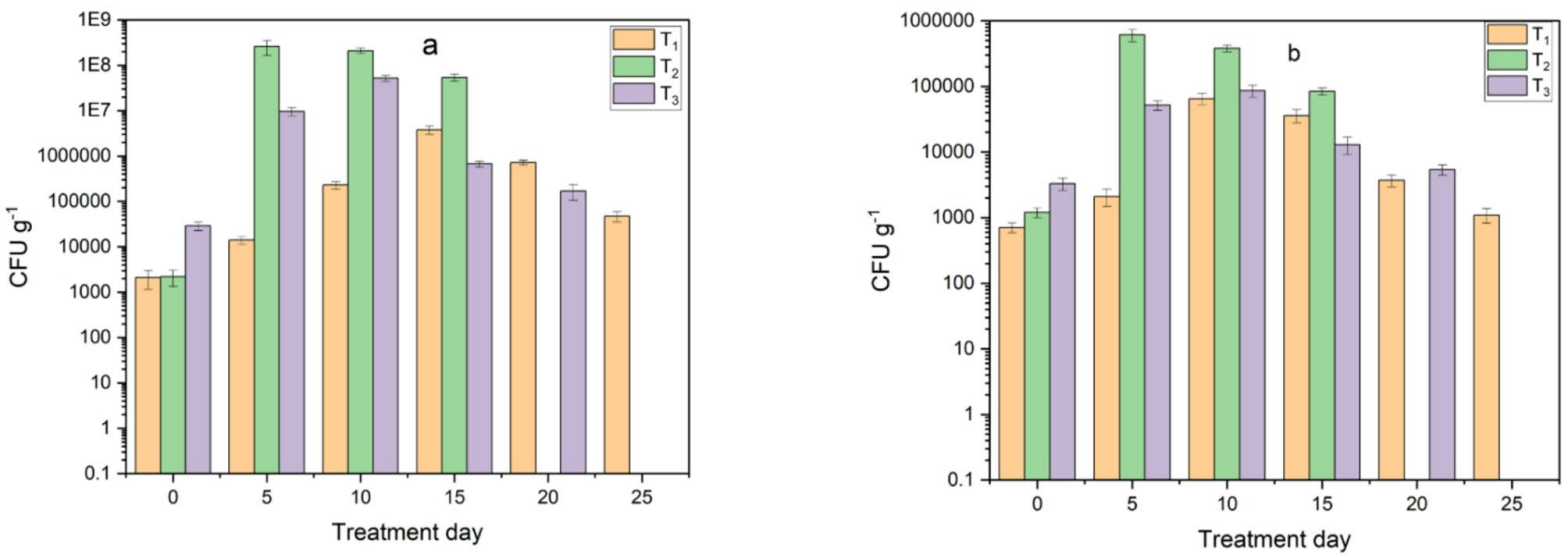
Monitoring of microbial load in treatments during composting after adding biochar: (**a**) total bacteria and (**b**) total fungi.

Microbiological analysis showed a significant variation in the total count of bacteria and fungi between treatments. The second treatment (T_2_) using used cooking oil showed the highest number of bacteria and fungi in the first and second weeks, with 3.1 × 10^7^ and 2.3 × 10^7^ CFU g^−1^ total bacteria and 9.1 × 10^4^ and 5.8 × 10^4^ CFU g^−1^ fungi, respectively. In third treatment (Inoculum), the numbers of bacteria and fungi were initially higher than in T_1_ and T_2_ treatments, reaching their highest value in the second week with 3.2 × 10^6^ CFU g^−1^ total bacteria and 1.6 × 10^4^ CFU g^−1^ fungi. However, the T_1_ (control) treatment showed the lowest number of microbes overall, as shown in Fig. 14. After adding biochar, the total count of bacteria and fungi were increased due to improved microbial activities and the rapid decomposition of waste, leading to an increase the number of microbes which agree with Ravindran et al.^27^ as shown in Fig. 15.

**Fig. 16:**
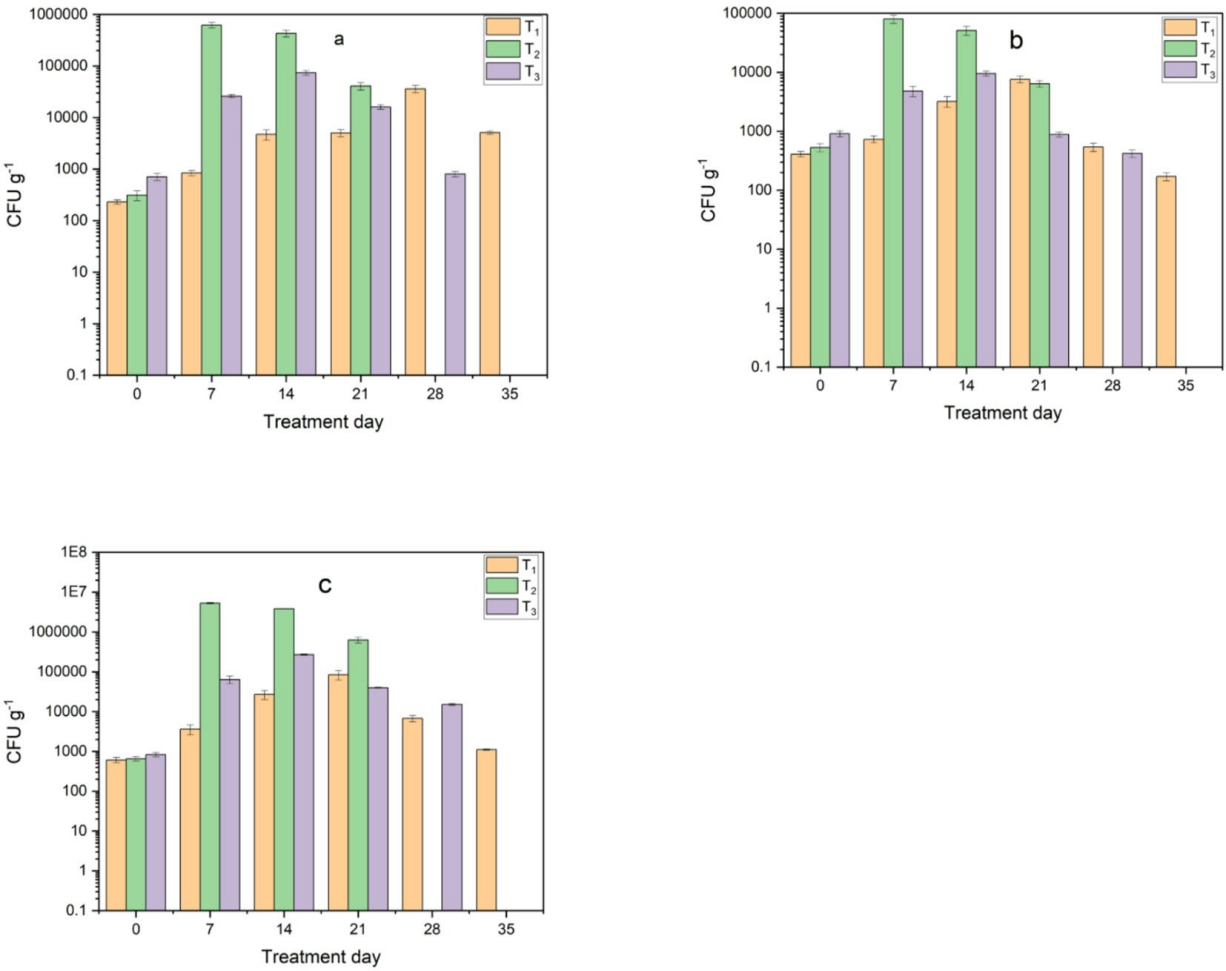
Monitoring of microbial load in treatments during composting before adding biochar: (**a**) actinomycetes, (**b**) cellulolytic bacteria and (**c**) proteolytic bacteria.

**Fig. 17:**
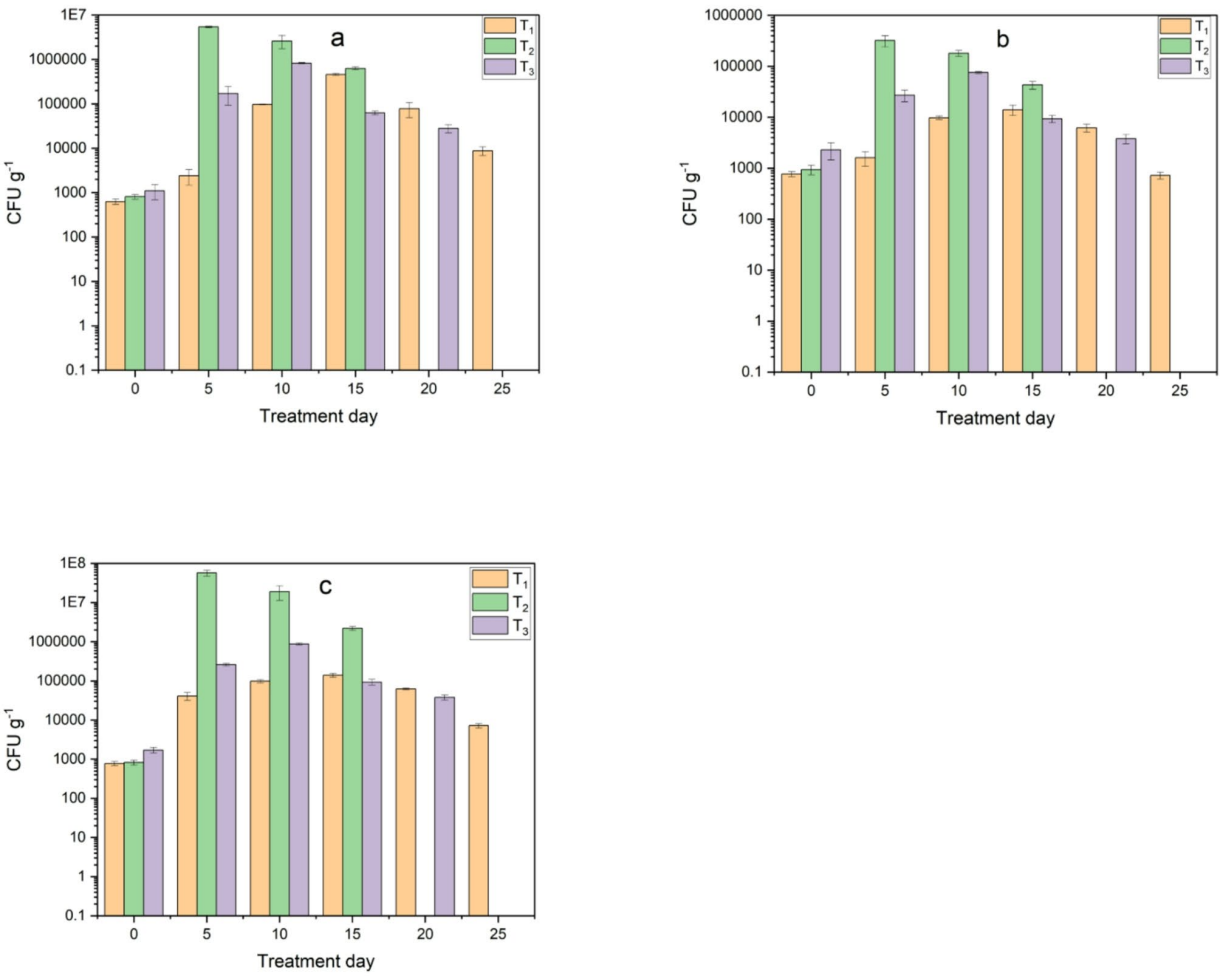
Monitoring of microbial load in treatments during composting after adding biochar: (**a**) actinomycetes, (**b**) cellulolytic bacteria and (**c**) proteolytic bacteria.

Results for the second treatment (T_2_) on the 7th day showed that the total actinomycetes, cellulolytic bacteria and proteolytic bacteria were 6.2 × 10^5^ CFU g^−1^, 8 × 10^4^ CFU g^−1^, 5.3 × 10^6^ CFU g^−1^, respectively. While third treatment (T_3_) on the 14th day showed that the total actinomycetes, cellulolytic bacteria and proteolytic bacteria were 7.4 × 10^4^ CFU g^−1^, 9.5 × 10^3^ CFU g^−1^, 2.7 × 10^5^ CFU g^−1^, respectively. As for the first treatment (T_1_), it had the highest value of 5 × 10^4^ CFU g^−1^ total actinomycetes, 7.6 × 10^3^ CFU g^−1^ cellulolytic bacteria, 8.4 × 10^4^ CFU g^−1^ proteolytic bacteria. After adding biochar, changes occurred in the number of microbes and their increase as shown in Figs. 16 and 17. This is due to the fact that biochar provides favorable environmental conditions for the process of microbial metabolism and reproduction, thus increasing microbial activity. This is consistent with studies that indicated that adding biochar to compost improves microbial activities, physical and chemical properties, decomposition and maturity which consistent with studies^18,34^. As for the decrease in the number of microbes, it is a result of the decrease in organic compounds and nutrients, while at the end of the composting process; it indicates the maturity and stability of the final compost.

**Fig. 18:**
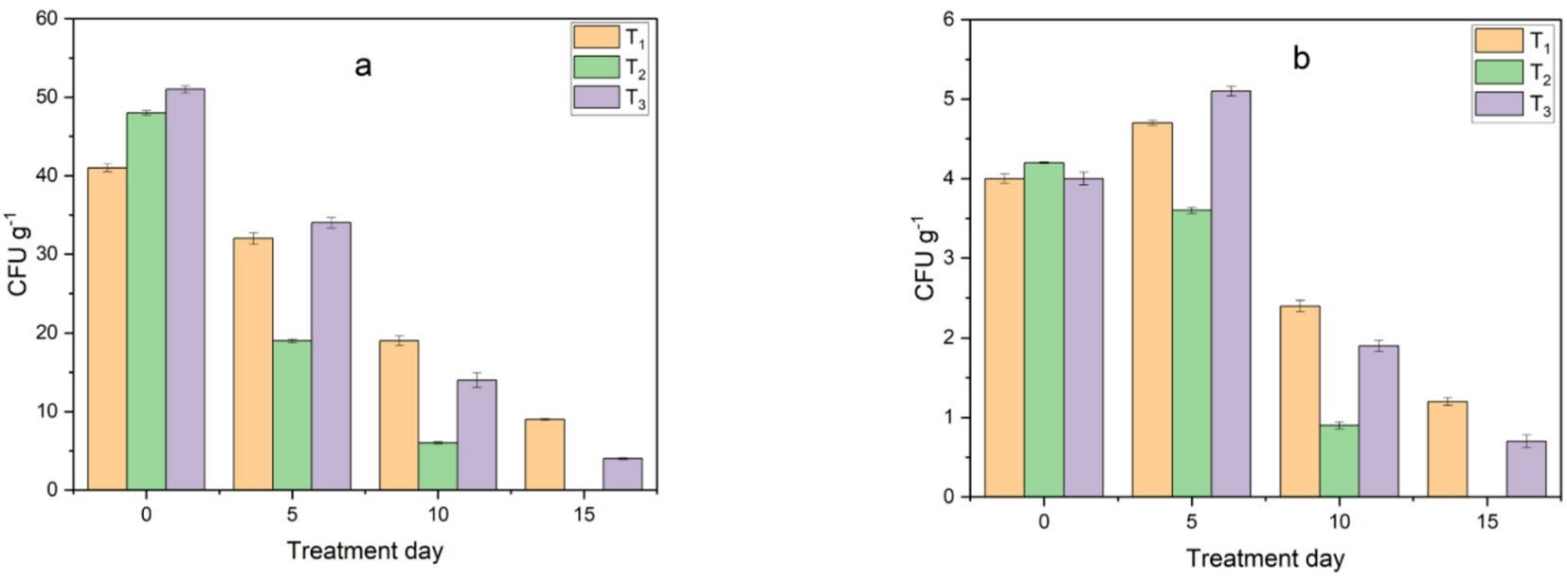
Monitoring of pathogenic bacteria load in treatments during composting before adding biochar: (**a**) *E. coli* and (**b**) salmonella.

**Fig. 19:**
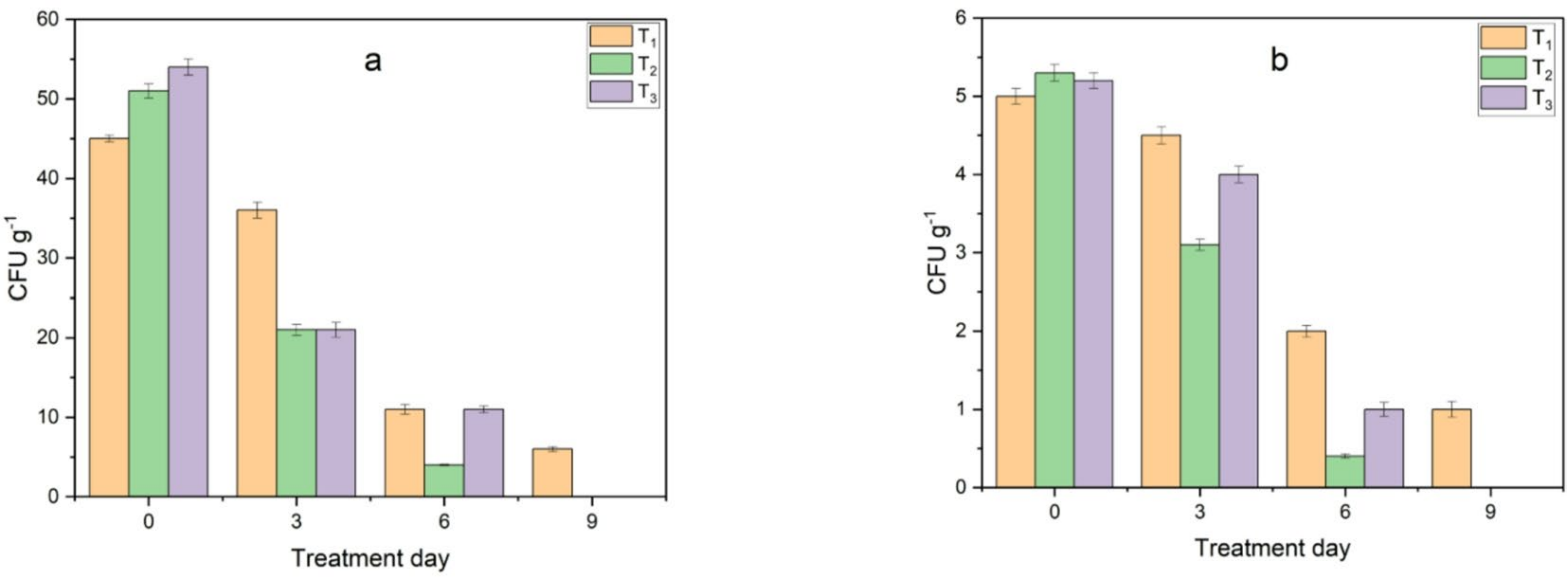
Monitoring of pathogenic bacteria load in treatments during composting after adding biochar: (**a**) *E. coli* and (**b**) salmonella.

Moreover, *E. coli* and Salmonella were investigated during the composting process. The pathogenic bacterial contaminants showed the highest results during the first week as shown in Fig. 18. However, after one week, they started to decrease. In some treatments they decreased from the third day and were all eliminated after two weeks or less as shown in Fig. 19. The elimination of pathogenic bacteria may have occurred due to the harsh environmental conditions created inside the bioreactor, such as high temperature, acidity and the addition of biochar, which changed the unfavorable nutrient conditions for the reproduction of these bacteria, which agree with^31^.

## Conclusion

The moisture content remained within the optimal range due to the balance between the water resulting from the respiration of microbes and the water lost with the exhaust gas. Increasing the amount of bioavailable carbon by adding used oil enhanced the biodegradation process resulting in a further reduction of the moisture content. The heat generated by the degradation of organic matter raised the temperature during the three thermal phases. The addition of the inoculum reduced the delay phase and increased the rate of temperature rise, while the addition of used oil accelerated the degradation of fat, proteins and complex carbohydrates, making more carbon biologically available to maintain longer periods of high temperatures. The composting process produced amounts of CO_2_ and CH_4_ emissions during the production process with the highest values obtained at the beginning of the experiment and gradually decreasing until the end. The addition of oil resulted in higher carbon dioxide (CO_2_) daily emissions compared to the rest of the treatments, indicating continuity and acceleration of the degradation process. Methane emissions decreased in all treatments and often reached zero due to maintaining moisture levels and continuous ventilation throughout the experiments. The addition of biochar preserved the moisture content, stimulated the metabolic activity of microorganisms and thus increased the biodegradation process. The temperature increased quickly and the thermophilic phase period increased, thereby reducing the maturity period of the compost as a result of the addition of biochar. Adding biochar addressed the issue of gas emissions, reducing the amount of emissions by 24–33%. There was no methane emission throughout the experiment period in all treatments due to the addition of biochar, maintenance of humidity, ventilation, and reduction of the anaerobic environment. Biochar helped conserve nitrogen, increase its percentage, and reduce its loss in the form of NH_3_ emissions. As a result of increasing the temperature after adding the used oil and biochar, microbial pathogens (E. coli and Salmonella) decreased and were eliminated within a week compared to the control treatment.

## Data Availability

The datasets generated and/or analyzed of the current study are available from the corresponding author on reasonable request.
